# A Chemoproteomic Approach to Elucidate the Mechanism of Action of 6-Azasteroids with Unique Activity in Mycobacteria

**DOI:** 10.1021/acsinfecdis.3c00296

**Published:** 2023-09-29

**Authors:** Joshua M. Werman, Yu-Ching Chen, Tianao Yuan, Xinxin Yang, Nicole S. Sampson

**Affiliations:** † Department of Chemistry, Stony Brook University, Stony Brook, New York 11794-3400, United States; ‡ Program in Biochemistry and Structural Biology, Stony Brook University, Stony Brook, New York 11794-5215, United States; § Department of Chemistry, University of Rochester, Rochester, New York 14627-0216, United States

**Keywords:** photoaffinity label, click chemistry, synergy, potentiator, oxidative stress, inhibitor

## Abstract

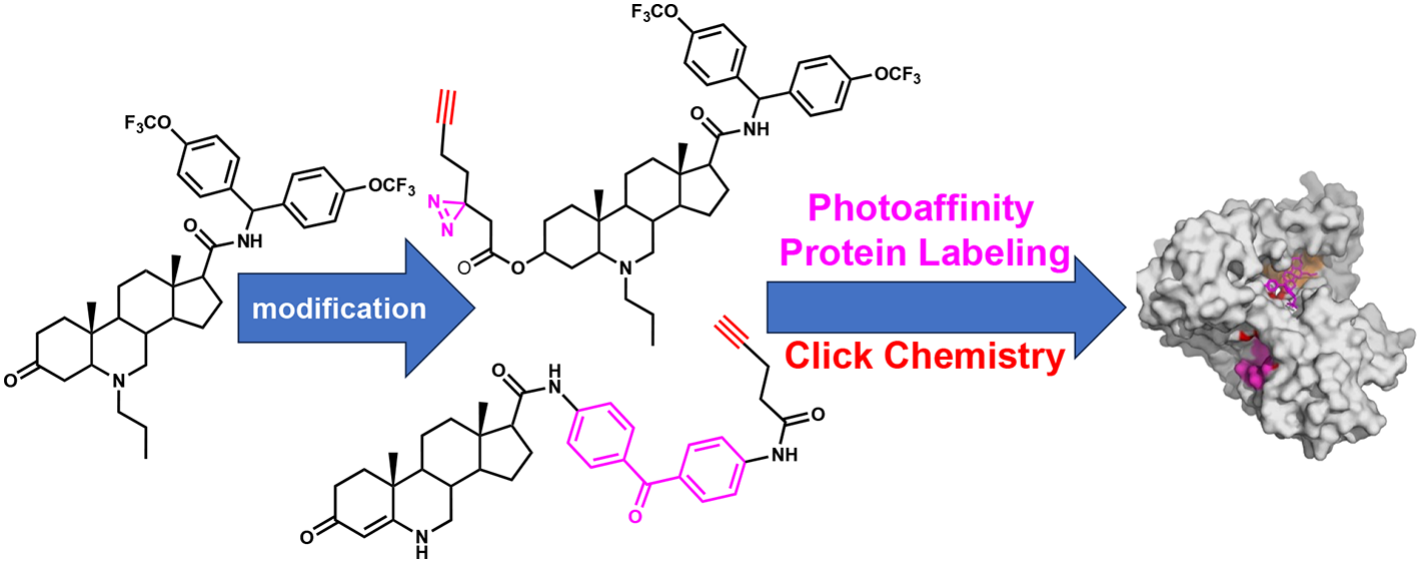

By illuminating key 6-azasteroid–protein interactions in both *Mycobacterium tuberculosis* (*Mtb*) and the closely related model organism *Mycobacterium marinum* (*Mm*), we sought to improve the antimycobacterial potency of 6-azasteroids and further our understanding of the mechanisms responsible for their potentiation of the antituberculosis drug bedaquiline. We selected a newly developed 6-azasteroid analog and an analog reported previously (*ACS Infect. Dis.***2019**, *5* (7), 1239–1251) to study their phenotypic effects on *Mtb* and *Mm*, both alone and in combination with bedaquiline. The 6-azasteroid analog, 17β-[*N*-(4-trifluoromethoxy-diphenylmethyl)carbamoyl]-6-propyl-azaandrostan-3-one, robustly potentiated bedaquiline-mediated antimycobacterial activity, with a nearly 8-fold reduction in *Mm* bedaquiline minimal inhibitory concentration (85 nM alone versus 11 nM with 20 μM 6-azasteroid). This analog displayed minimal inhibitory activity against recombinant mycobacterial 3β-hydroxysteroid dehydrogenase, a previously identified target of several 6-azasteroids. Dose-dependent potentiation of bedaquiline by this analog reduced mycobacterial intracellular ATP levels and impeded the ability of *Mtb* to neutralize exogenous oxidative stress in culture. We developed two 6-azasteroid photoaffinity probes to investigate azasteroid–protein interactions in *Mm* whole cells. Using bottom-up mass spectrometric profiling of the cross-linked proteins, we identified eight potential *Mm*/*Mtb* protein targets for 6-azasteroids. The nature of these potential targets indicates that proteins related to oxidative stress resistance play a key role in the BDQ-potentiating activity of azasteroids and highlights the potential impact of inhibition of these targets on the generation of drug sensitivity.

Tuberculosis (TB), a result of infection by the pathogen *Mycobacterium tuberculosis* (*Mtb*), is an ancient endemic disease that has managed to elude eradication by modern medicine. TB presents an enormous global burden; in 2022, it was the second most deadly infectious disease, after COVID-19.^1^ TB disproportionately affects the developing world, because *Mtb* is an opportunistic pathogen that spreads easily under conditions of poverty, overcrowding, and malnutrition. Although rates of TB transmission are slowly decreasing in regions where the disease is pervasive, a new threat looms: TB that is resistant to traditional frontline therapies, referred to as multidrug-resistant TB (MDR-TB). MDR-TB reduces the number of treatment options available, and worse overall outcomes are observed with MDR-TB. Globally in 2018, only 59% of patients with MDR-TB experienced favorable outcomes compared to 86% of patients that started on first-line TB treatment.^1^ Thus, there is still an unmet need to develop improved treatment regimens to shorten treatment duration, prevent relapse and resistance, and improve outcomes. Despite continued interest in the discovery of new TB drugs and the ever-expanding clinical and preclinical pipelines, only a few new TB drugs have been approved over the past five decades. Furthermore, many clinical and preclinical anti-TB drugs have redundant mechanisms of action, targeting ATP synthesis, protein synthesis, redox stress generation, DNA replication, or cell wall synthesis.^2^ Antibiotics belonging to the same mechanistic family are susceptible to cross-resistance and thus may not be useful for addressing complications resulting from resistant TB strains.^3^ TB therapeutics that take advantage of well-validated and novel biological pathways are integral to addressing the global TB problem. Furthermore, therapeutics that function synergistically with current anti-TB treatments are positioned to address MDR-TB by more rapidly and effectively clearing mycobacterial infection, reducing the time during which resistant mutants can arise.^4^

The discovery of new TB antibiotics with mechanisms outside those commonly targeted is no simple feat, in part because *Mtb* is readily adaptable. It maintains the capacity to reside in diverse environments in hosts, in either active or latent replicative states, making many classically indispensable biological pathways dispensable. To date, most of the anti-TB drugs that are newly approved or under clinical testing were identified from whole-cell-based approaches.^2^ Such approaches provide little in the way of mechanistic insight, and therefore, upon the discovery of compounds with antimycobacterial activity, further study is required to elucidate the mechanism of action.^5^

We previously reported the utility of a series of 6-azasteroids as adjuvants to frontline anti-TB therapeutics, a promising strategy to combat innate drug resistance in *Mtb*. These compounds were originally hypothesized to target cholesterol metabolism, but we previously uncovered associations between 6-azasteroid activity and several non-cholesterol-metabolizing pathways, including the Mce3R-regulated oxidative stress pathway, which is required for 6-azasteroid activity.^6^

In the study described in this work, we further explored the phenotypic effects of several 6-azasteroids alone and in combination with the ATP synthase inhibitor bedaquiline (BDQ) to better understand the mechanisms of potentiation of BDQ by 6-azasteroids. Furthermore, we sought to directly characterize the biomolecular interactions of 6-azasteroids by leveraging two photoactivatable cross-linking probes that would enable the labeling, enrichment, and proteomic analysis of the molecular target(s) of 6-azasteroids. Although previous attempts to untangle the azasteroid mechanisms of action have not produced conclusive results, we reasoned that a direct-interaction assay had the potential to identify proteins that contribute to azasteroid activity. Using the two probes, we identified eight proteins that mediate responses to oxidative stress in mycothiol-related pathways, as well as in the electron transport chain. The potential of these proteins to be targets of 6-azasteroids was corroborated by computational molecular docking studies and a substantial amount of experimental evidence from the literature. The potential polypharmacological properties of 6-azasteroids make it an attractive chemical class for further development as anti-TB agents.

## Results

### Screening of 6-Azasteroid Analogs for Activity in *Mtb* and *Mycobacterium marinum*

**Figure 1:**
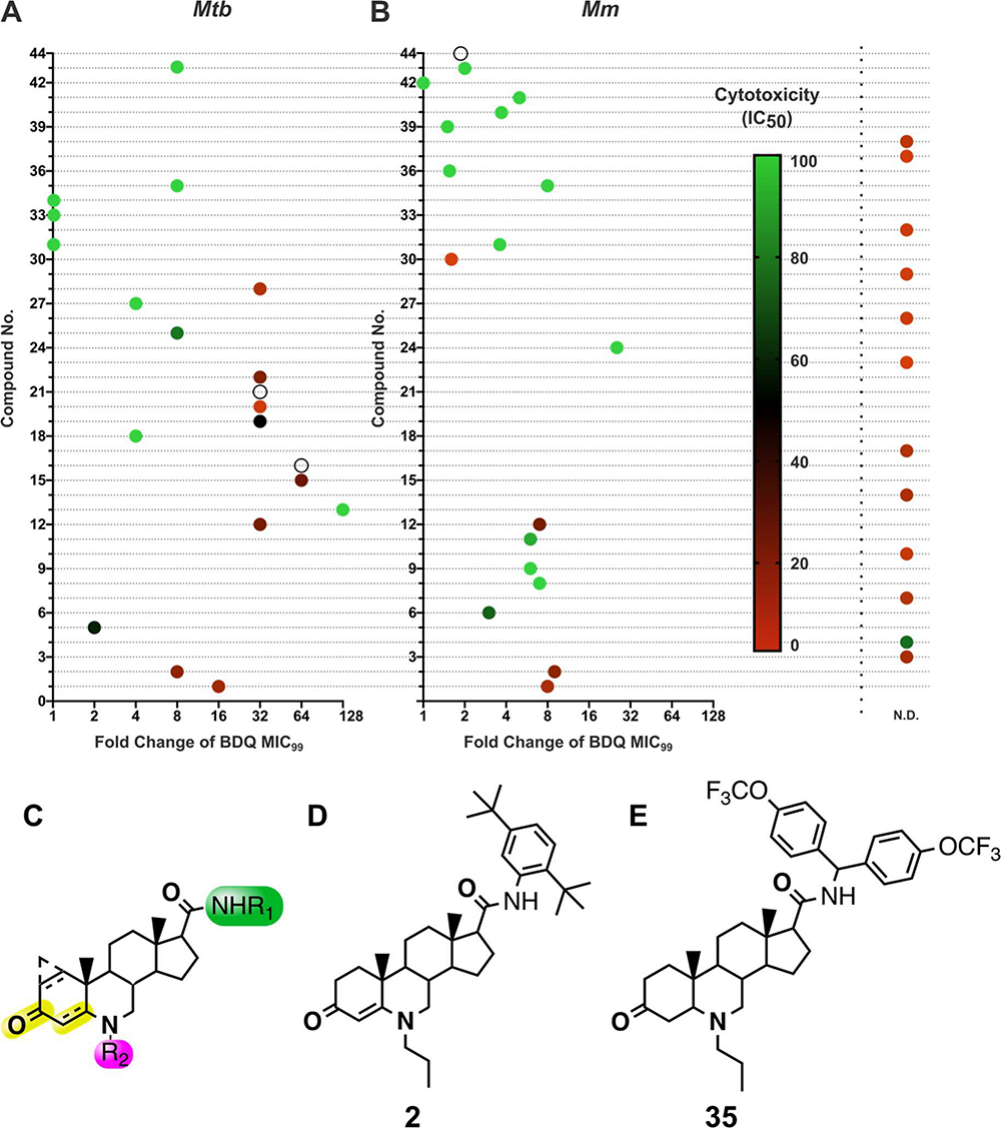
6-Azasteroid scaffold and overview of the 6-azasteroid analog activity. (A, B) 6-Azasteroid activity against *Mycobacterium tuberculosis* (*Mtb*) and *Mycobacterium marinum* (*Mm*) and cytotoxicity to HepG2 and THP-1 cells. For potentiation assays, *Mm* and *Mtb* were cultured separately in Middlebrook 7H9 broth supplemented with bovine serum albumin and glycerol as the sole carbon source. Growth inhibition was measured by visual inspection or by staining with an Alamar Blue reagent. The fold change in bedaquiline (BDQ) minimal inhibitory concentration (MIC_99_) upon azasteroid cotreatment is relative to the MIC_99_ of BDQ without azasteroid cotreatment. For cytotoxicity assays, HepG2 and THP-1 cells were grown in Minimum Eagle Medium and Roswell Park Memorial Institute medium, respectively; and cell viability was measured by staining with Alamar Blue reagent. N.D., BDQ co-MIC not determined. Open circle, cytotoxicity not determined. (C) 6-Azasteroid scaffold and sites of structural modifications indicated in color. (D) Structure of **2**. (E) Structure of **35**.

We previously demonstrated that 6-azasteroid **2**, which has a (2,5-di-*tert*-butyl)anilide group at R_1_ and propyl group at R_2_, potentiates the activity of isoniazid and BDQ against both *Mtb* and *Mycobacterium marinum* (*Mm*) by 8-fold under normoxic conditions.^6^ In the work described herein, guided by activity measurements, we synthesized a small library of 6-azasteroid analogs with an R_1_ side chain with a single anilide group, regardless of R_2_ or C4–C5 saturation. A second group of analogs contains a (diphenylmethyl)carbamoyl R_1_ side chain, a saturated or unsaturated A ring (C4–C5), and a hydrogen or a propyl group at R_2_. Additional analogs contain either a (4-trifluoromethoxy-diphenylmethyl)carbamoyl or a benzophenone R_1_ side chain, a saturated or unsaturated A ring (C4–C5), and various modifications at R_2_ (Figures 1C and S1).

The antimicrobial activities of the synthesized 6-azasteroids were determined at a fixed concentration of 20 μM and serial dilution of BDQ, and growth inhibition was measured by Alamar Blue staining or visual inspection in microbroth assays. Efficacy is reported as the fold-change reduction of BDQ minimal inhibitory concentration (MIC_99_) in the presence of the 6-azasteroid relative to that in its absence (Figure 1A, 1B). In addition, the metabolic viabilities of cultured HepG2 and THP1 cells after incubation for 72 h with individual 6-azasteroids were determined by Alamar Blue staining to investigate the cytotoxicity of the analogs to eukaryotes (Figure 1A, 1B). 6-Azasteroid antimycobacterial activity was improved through iterative medicinal chemistry efforts guided by analysis of structure–activity relationships. We observed that compounds with an ortho-functionalized (diphenylmethyl)carbamoyl side chain at R_1_ displayed impressive BDQ potentiation and low cytotoxicity, leading to a wide therapeutic window. Activity was also influenced by the saturation state of the A ring and the functionality at the 6-position nitrogen. Although most of the 6-azasteroids with an unsaturated A ring had moderate cytotoxicity (generally with an IC_50_ between 20 and 80 μM), saturated-A-ring azasteroids showed low-micromolar cytotoxicity. The nature of the substitution at the 6-position nitrogen affected cytotoxicity. For example, acylation ablated both activity and cytotoxicity, but propylation effectively tempered cytotoxicity while maintaining activity. We designated compound **35** as a lead compound because of its large therapeutic window and impressive potentiation of BDQ, and we compared it with **2** (Figure 1D, 1E). At 20 μM, **2** and **35** displayed greater than 8-fold reductions in BDQ MIC_99_, and their IC_50_ values in mammalian cells were 25 and >100 μM, respectively. Subsequent phenotypic experiments were conducted with **2** and **35**.

### Inhibition of Mycobacterial 3β-Hydroxysteroid Dehydrogenase by 6-Azasteroids

6-Azasteroids inhibit both human adrenal 3β-hydroxysteroid dehydrogenase (3β-Hsd)^7^ and mycobacterial 3β-Hsd,^8^ which shares less than 40% identity with its mammalian counterpart. Considering these observations, 6-azasteroids were screened for potentiation activity with isoniazid and BDQ against *Mtb* 3β-Hsd knockout mutants, and potentiation activity was found to be maintained, indicating that 3β-Hsd is not a key target of 6-azasteroids.^6^ More recently, 6-azasteroids have been shown to inhibit *Mycobacterium leprae*,^9^ which has no cholesterol catabolism genes other than a gene for a 3β-Hsd homologue that shares greater than 75% amino acid identity with *Mtb* 3β-Hsd.

Given the conflicting evidence for the role of 6-azasteroids in inhibition of *Mtb* 3β-Hsd, we screened several 6-azasteroids (at 50 μM) for inhibitory activity against recombinant *Mtb* 3β-Hsd with dehydroepiandrosterone as a substrate fixed at a concentration 1.8 times *K*_m_ and NAD^+^ at a fixed concentration 1.75 times *K*_m_.^10^ The rates of dehydroepiandrosterone dehydrogenation were determined with or without inhibitor by monitoring the NADH formation at 340 nm. Inhibition percentages were determined from the ratios of the rates of dehydrogenation of dehydroepiandrosterone to androstenedione with inhibitor versus the rates without inhibitor.

**Figure 2:**
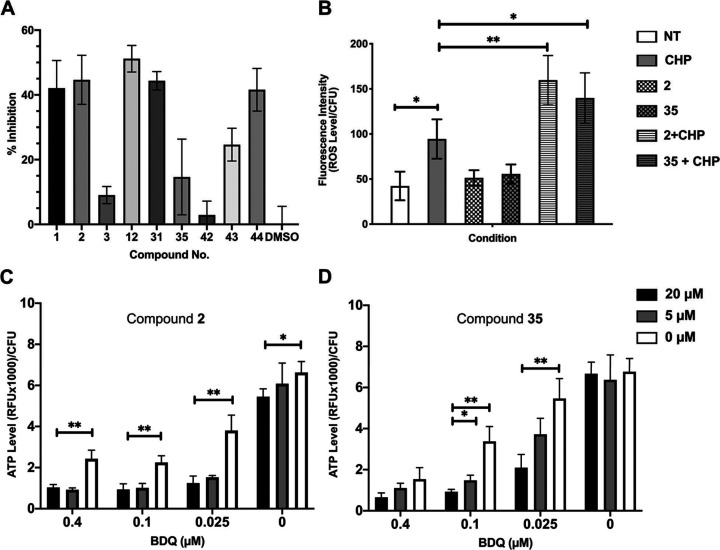
6-Azasteroid phenotypic data. (A) Inhibition of recombinant *Mtb* 3β-hydroxysteroid dehydrogenase. Inhibition by the 6-azasteroids was determined at a 1 μM enzyme concentration in the presence of 50 μM of each 6-azasteroid. The concentration of NAD^+^ was held fixed at 350 μM, and that of dehydroepiandrosterone was held fixed at 220 μM.^8^ (B) 6-Azasteroid impact on reactive oxygen species (ROS). *Mtb* (CDC1551) cultures grown to mid logarithmic phase (OD_600_ = 0.6) were pretreated with **2** or **35** (20 μM) for 6 h. Then the cells were washed to remove excess 6-azasteroid and treated with 5 mM cumene hydroperoxide (CHP) for 30 min. Oxidative stress was quantified and normalized to colony forming unit (CFU) counts. NT, no treatment. (C, D) Impact of 6-azasteroids **2** (C) and **35** (D) on bedaquiline (BDQ)–mediated inhibition of ATP production. RFU, relative fluorescence units. Error bars indicate standard deviation of the mean of three replicates; *p < 0.05; **p < 0.01.

Compounds with a saturated A ring generally showed lower inhibitory activity than compounds with an unsaturated A ring, whereas there was no direct correlation between activity and alkylation of the 6-position nitrogen or replacement of an anilide side chain with a (diphenylmethyl)carbamoyl side chain (Figure 2A). Lead compound **35** and its derivative (compound **51**) showed insignificant inhibition of 3β-Hsd activity, a result that is consistent with prior observations that 6-azasteroid potentiation of BDQ is unrelated to 3β-Hsd inhibition.

### Impact of 6-Azasteroids **2** and **35** on Reactive Oxygen Species in *Mtb* and *Mm*

We previously established that azasteroid activity requires the Mce3R regulon, which has been implicated in *Mtb* persistence in macrophages through resistance to oxidative stress.^6^ To better understand the relationship between azasteroids and oxidative stress, we measured the effects of treatment with **2** or **35** on the generation of reactive oxygen species (ROS) in the presence and absence of peroxide stress in *Mtb.* Specifically, we measured the ROS levels upon treatment of wild-type (WT) *Mtb* cultures with 5 mM organic peroxide, cumene hydroperoxide (CHP), for 30 min with or without preincubation of the cultures with 6-azasteroid **2** or **35** for 6 h. We found that 6-azasteroid treatment alone had a negligible effect on intracellular ROS levels (Figure 2B). In comparison, WT *Mtb* pretreated with the 6-azasteroids was sensitized to oxidative stress generated by the addition of exogenous CHP. Under these conditions, 6-azasteroid pretreatment resulted in a greater accumulation of intracellular oxidants than that observed in mycobacteria that had not been pretreated with a 6-azasteroid. These data show that the 6-azasteroids interfered with the ability of *Mtb* to neutralize oxidative stress in vitro.

### Impact of 6-Azasteroids **2** and **35** on BDQ-Mediated Inhibition of ATP Production

The primary mechanism of BDQ antimycobacterial activity is targeting of the ε subunit of ATP synthase, resulting in decreased ATP production. To assess BDQ potentiation by 6-azasteroids **2** and **35**, we assayed intracellular ATP levels under cotreatment as a function of BDQ concentration. CDC1551 *Mtb* culture grown to OD_600_ = 0.6 in 7H9 glycerol-containing media was exposed to 0.4, 0.1, 0.025, or 0 μM BDQ in combination with 20, 5, or 0 μM **2** or **35** for 96 h; and then ATP levels were measured (Figure 2C,D). ATP production was not greatly affected by either **2** or **35** alone, indicating that their activity is likely unrelated to direct interaction with ATP synthase. When **2** or **35** was administered in combination with BDQ, a dose-dependent reduction in the ATP concentration in the *Mtb* CDC1551 cultures was observed: specifically, the cellular ATP concentration was reduced to less than 50% of that seen upon treatment with BDQ. This effect was observed for both 6-azasteroids at concentrations of 5 and 20 μM.

### Identification of 6-Azasteroid Protein Targets

**Figure 3:**
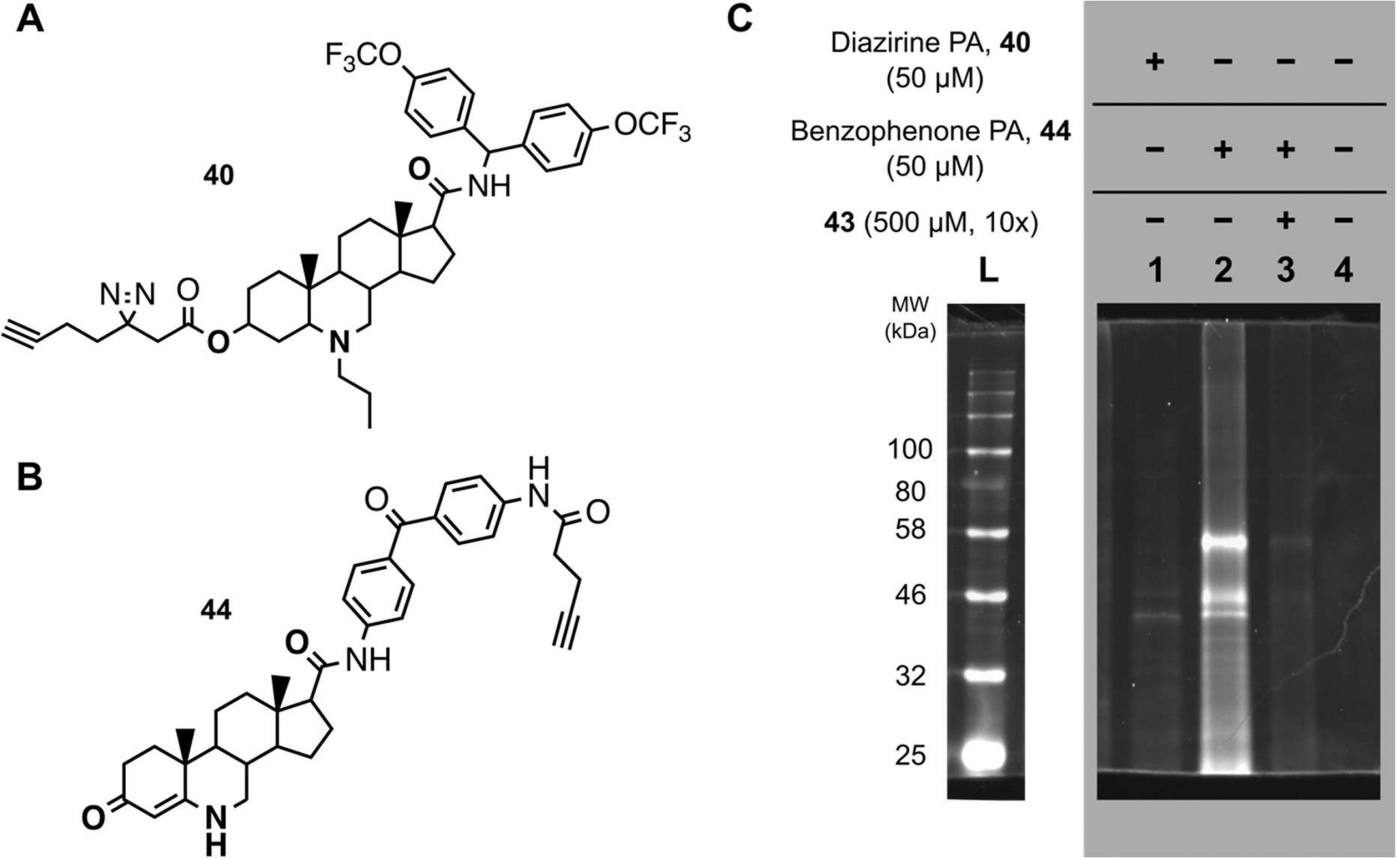
Whole-cell lysate interaction assay, Azido-PEG3-TAMRA-Biotin enrichment. (A) Compound **40** (Diazirine PA) and (B) compound **44** (Benzophenone PA). The nonprobe inhibitor **43** is shown in Figure S2. (C) Fluorescence image of SDS-PAGE gel–*Mm* lysates incubated with **40** (lane 1), **44** (lane 2), **44** plus excess **43** (lane 3), or DMSO (lane 4) after click conjugation with azido-PEG3-TAMRA-biotin and streptavidin–agarose-bead-mediated enrichment. The ladder (**L**) is shown separated from the fluorescence gel to depict only the enriched samples and not the samples visualized directly after the click reaction; the full image can be found in the Supporting Information (Figure S3B).

To identify potential protein targets of the 6-azasteroids, we developed two 6-azasteroid photochemical probes that contain a terminal alkyne moiety for covalent attachment of affinity tags to enable the isolation of target proteins, as well as a diazirine or benzophenone photoreactive cross-linker moiety to enable covalent attachment to protein targets upon irradiation with UV light.^11^ Like their parent compounds, the two hydrophobic probes, designated Diazirine PA (**40**, Figure 3A) and Benzophenone PA (**44**, Figures 3B), showed BDQ potentiation activity in *Mm* (Figure 1B). The diazirine moiety was attached to the azasteroid scaffold through a short linker to the A ring, and the benzophenone moiety was attached as a side chain on the D ring. The location of photo-cross-linking moieties on opposite hemispheres of the two probes was a crucial feature of the experimental design that favored elimination of proteins that bound nonspecifically to the probes from our downstream data analyses. A terminal alkyne adjacent to the cross-linking functional groups allowed the attachment of an affinity tag by means of azide–alkyne click chemistry.

We employed protein-enrichment strategies that involved copper-catalyzed azide–alkyne cycloaddition (CuAAC) click reactions with azido-PEG3-biotin or azido-PEG3-TAMRA-biotin reagents for streptavidin capture and visualization by streptavidin-conjugated horseradish peroxidase blots or direct fluorescence imaging of SDS-PAGE gels.

For proteomic analysis, we utilized CuAAC click reactions with azido-functionalized agarose beads or azido-functionalized magnetic nanoparticles (which eliminated the need for streptavidin and thus the accompanying background of natively biotinylated proteins), in addition to streptavidin-functionalized magnetic nanoparticles to ensure consistency between proteomic and Western blot methodologies.

In brief, 6-azasteroids or control probes were incubated with *Mm* whole cells, which were then washed, irradiated with UV light (365 nm), and lysed. Lysates were subjected to click reaction conditions with one of the aforementioned azido reagents, and the products were analyzed by Western blotting or direct fluorescence imaging of SDS-PAGE, or used directly for proteomic studies.

By visual analysis of Western blots and SDS-PAGE fluorescence images resulting from pull-down interaction studies, we observed two enriched protein bands of interest in the molecular weight range of 35–44 kDa and one enriched protein band of interest in the range of 48–55 kDa (Figure 3C). In results that were validated by replicate studies, the proteins between 35 and 44 kDa and between 48 and 55 kDa were captured by both Diazirine PA and Benzophenone PA but were not captured in the DMSO control sample or when protein capture by **44** was challenged by competition with a 10-fold excess of **43** (Figure S2), an analog of **44** that is not functionalized with a terminal alkyne linker and cannot participate in the CuAAC click reactions required to enrich and visualize interacting proteins (Figure 3). The molecular weight ranges indicated above were used to filter proteins identified in subsequent proteomic analyses.

We initially detected a large number of proteins across the three different enrichment strategies, a result that was expected given the high potential for nonspecific interactions related to reactive carbene-intermediate-based photo-cross-linkers and nonspecific protein binding by the bead matrices utilized to immobilize the captured proteins. We identified a pool of 494 unique *Mm* proteins (only 289 of which had *Mtb* orthologs) that were enriched in at least one data group for either the diazirine-based probe or the benzophenone-based probe and that fell in the molecular weight ranges determined by means of fluorescence visualization of captured proteins (Figure 3C). From a functional perspective, when characterized by KEGG pathway ID (KEGG = Kyoto Encyclopedia of Genes and Genomes), the pool of captured proteins showed no significant functional pathway enrichment relative to the makeup of the *Mm* proteome, indicating a lack of apparent functional biases under the experimental conditions (Figure S4A,B).

Hit candidates were prioritized by identification of proteins under multiple enrichment conditions across two biological replicates (Figure S5). We further filtered the 494 *Mm* hit proteins by including only proteins with *Mtb* orthologs or proteins with >75% amino acid sequence similarity in subsequent analyses. In this way, 77 unique prioritized proteins were identified across both probes. To further filter these proteins, we mined data from validated mycobacterial platforms that elucidated gene–drug sensitivity. Li et al. employed a genome-wide CRISPR interference library to identify genes mediating drug potency in the presence of nine different anti-TB drugs.^12^ We leveraged this data to elucidate which of our hit proteins conferred sensitivity to three drugs—BDQ, isoniazid, and rifampicin—that show enhanced activity in the presence of 6-azasteroids.^6^

Using our two probes, we identified eight proteins that were enriched in proteomic analyses; that caused sensitivity to BDQ, isoniazid, and rifampicin when the gene that coded for the protein was knocked down by CRISPR interference (Figures S6 and S7); and that were within the molecular weight ranges identified by fluorescence gel imaging (Figure S8).

The identified proteins have a variety of functions, which are described in Table 1. We selected several proteins for further study that have been annotated to catalyze antioxidant-related functions: Rv2855 (Mtr), Rv3913 (TrxB2), and Rv1623c (CydA). Antioxidant biological pathways, namely Mce3R-regulated genes,^6^ have previously been shown to be affected by azasteroids, warranting the emphasis on the three highlighted antioxidant related targets. The remaining five proteins have functions related to cell wall biosynthesis, drug detoxification, protein translocation, and virulence. These five proteins are worthy of follow-up, but because of the connection to antioxidant function, we only performed docking studies with the three antioxidant-related targets. To assess the validity of the hits identified by proteomic analysis, we performed *in silico* molecular docking studies with each of these three proteins using two affinity probes (**40** and **44)** and the azasteroid analogs from which they were derived (**35** and **43**) (Figures S8, S9, and S10).

**Table 1:** Overview of Protein Hits from Proteomic Analyses of 6-Azasteroid Interaction Assays

Rv no.	MMAR ID	gene	description	molecular weight (kDa)
Rv2586c	MMAR_2121	*secF*	Involved in protein export, part of the prokaryotic protein translocation apparatus	47.7
Rv2610c	MMAR_2092	*pimA*	Involved in the first mannosylation step in phosphatidylinositol mannoside biosynthesis (transfer of mannose residues onto phosphatidylinositol, leading to the synthesis of phosphatidylinositol monomannoside)	40.2
Rv1477	MMAR_2284	*ripA*	Peptidoglycan hydrolase supposed to be involved in virulence	49.5
Rv2855	MMAR_1875	*mtr*	Involved in reduction of mycothiol	49.5
Rv3913	MMAR_5477	*trxB2*	Catalyzes disulfide reduction by pyridine nucleotides through an enzyme disulfide and a flavin, seems to be regulated by sigH (Rv3223c product)	35.2
Rv3617	MMAR_5114	*ephA*	Biotransformation enzyme that catalyzes hydrolysis of epoxides (alkene oxides, oxiranes) to less reactive and more water-soluble dihydrodiols by the trans addition of water, thought to be involved in detoxification reactions	35.6
Rv3194c	MMAR_1370	*Rv3194c*	Serine protease important to pathogen-associated virulence factors involved in invasion, persistence, and degradation of host defense	35.3
Rv1623c	MMAR_2426	*cydA*	Involved in the terminal step of the respiratory chain (i.e., aerobic respiration)	54.2

### *In Silico* Molecular Docking Studies

We performed *in silico* molecular docking studies using AutoDock Vina software (version 1.2.0) to model protein–azasteroid interactions. We screened the azasteroids by placing the search box over binding sites that were determined by homology modeling or by P2Rank (https://prankweb.cz/), a tool for predicting protein–ligand binding sites and acquired calculated affinities for each protein–ligand pair (Table 2).^13,14^

**Table 2:** Binding Affinities of 6-Azasteroids **35** and **43** for Hit Proteins, Calculated Using AutoDock Vina^a^

	TrxB2 (Rv3913)		Mtr (Rv2855)		CydA (Rv1623c)	
protein	**35**	**43**	**35**	**43**	**35**	**43**
Binding affinity (Δ*E*, kcal/mol)	–8.7	–11.3	–10.0	–10.0	–10.8	–11.9
Binding site	NADP^+^ site	Blocking NADP^+^ site	FAD groove	FAD groove	MK9 binding site	MK9 binding site

a Abbreviations: FAD, flavin adenine dinucleotide; MK9, menaquinone-9.

**Figure 4:**
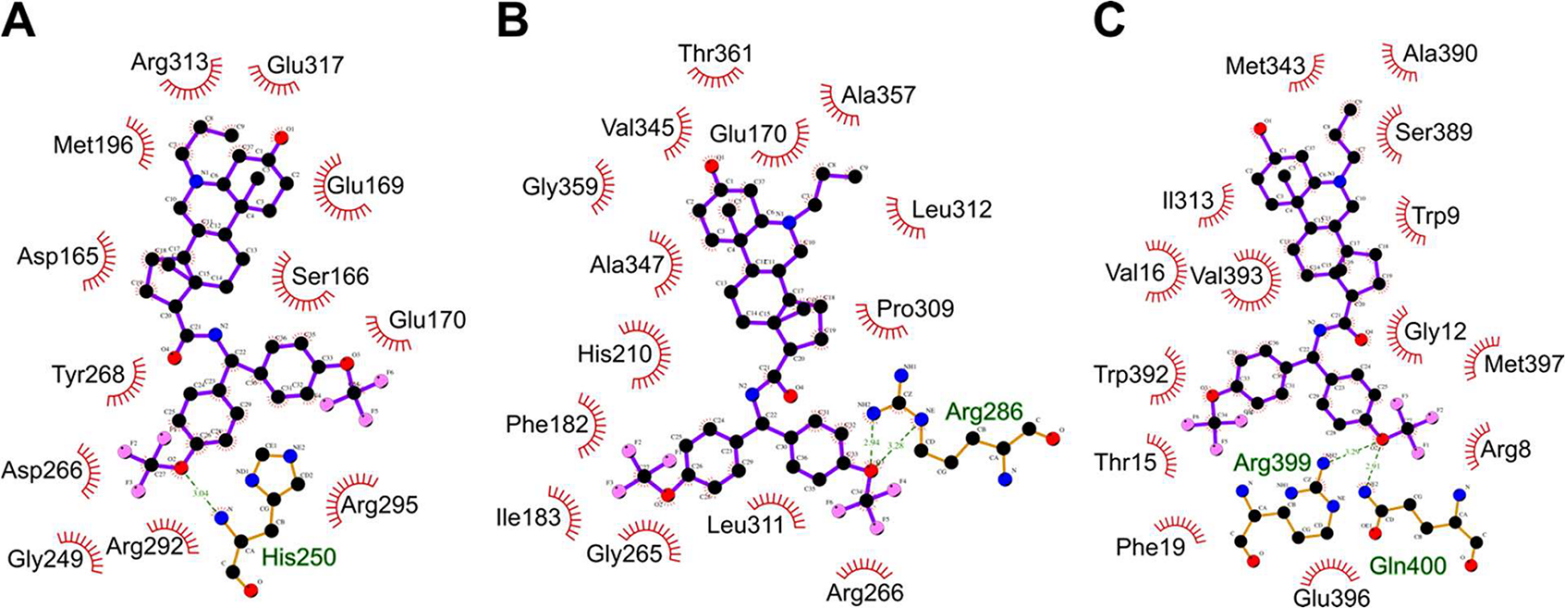
Binding poses of 6-azasteroid 35 in hit protein structures, calculated using AutoDock Vina and LigPlot+. Compound docking in the binding site as determined by homology modeling or with the binding-site software P2Rank: (A) TrxB2 (Rv3913), (B) Mtr (Rv2855), and (C) CydA (Rv1623c). Binding poses are visualized in 2D using LigPlot+ software.

The docking studies revealed that the azasteroids categorically fit within the binding sites of the hit proteins. Figure 4 depicts the lowest energy states of **35** docked into three of the prioritized hit protein structures. In each simulation, the ligand is docked within the cavity predicted as the most probable binding pocket by P2Rank software.

The simulation showed that **35** binds to TrxB2 by partially blocking the NADP^+^ binding site. In the simulation involving the cocrystal structure of TrxB2 with Mg^2+^, NADP^+^, and flavin adenine dinucleotide (PDB: 2A87), **35** blocks the interaction of NADP^+^ with the metal cofactor and occupies a portion of the proposed binding channel for mycothiol, potentially preventing the interaction of mycothiol with the catalytic site. Key residues involved in NADP^+^ binding include S166 (potential H-bonding to the side chain amide), Y268 (potential H-bonding to one of the trifluoromethoxy groups), and R295 (potential H-bonding to the other trifluoromethoxy group) (Figure 4A).

In our analysis of the interaction of **35** with Mtr, we aligned a homology modeled Mtr structure from AlphaFold (AF-P9WHH3-F1) with the structure of a glutathione reductase from *Escherichia coli* (PDB: 1GET) cocrystallized with NADP^+^ and flavin adenine dinucleotide, to determine the binding poses of the endogenous ligands in Mtr. The amino acid sequences of the two enzymes showed 92% sequence coverage and 30% sequence identity, and both secondary and tertiary structural homology were high. The side chain of **35** overlapped with the electron density corresponding to the nicotinamide moiety of NADP^+^, indicating that 6-azasteroids likely prevent electron donation mediated by the nicotinamide cofactor. The side chain of **35** forms close contacts with F182 (π–π interaction), I183 (van der Waals interaction), and shows potential for interaction with R286 (H-bonding) (Figure 4B).

Compound **35** did not exhibit close contacts with the conserved NADP^+^-binding residues in either TrxB2 or Mtr, thereby indicating that the binding interactions between **35** and the enzymes are attributable to nonconserved residues unique to each enzyme.

Aurachin D, an experimentally validated inhibitor of CydA, has been modeled to bind in the space between the catalytic heme and menaquinone-9, interacting with W9 and R8, thereby preventing electron transfer and inhibiting the enzyme.^15^ When **35** was docked, its steroid scaffold overlapped with menaquinone-9, and the side chain of **35** protruded deep into the binding pocket and occupied a location similar to that occupied by aurachin D (Figure 4C).

## Discussion

In this study, we explored the phenotypic effects and mechanism of antimycobacterial 6-azasteroids. Developed by means of drug optimization efforts, lead compound **35** strongly potentiated BDQ, reducing its MIC_99_ from 85 to 11 nM, and showed no signs of toxicity at up to 100 μM in two mammalian cell lines. Previous attempts to elucidate the mechanism of action of 6-azasteroids did not yield direct drug interactors. Therefore, we sought to characterize the activity of azasteroids phenotypically and by chemical-interaction assays.

6-Azasteroids have previously been shown to have affinity for mammalian 3β-Hsd^8^ and, more recently, have been proposed to inhibit the *M. leprae* 3β-Hsd ortholog,^9^ which shares over 75% sequence similarity with the mammalian enzyme. In *M. leprae,* 6-azasteroids inhibit the dehydrogenation of cholesterol to cholestenone and the concomitant reduction of NAD^+^ to generate NADH.^9^ This dehydrogenation reaction is hypothesized to be a key pathway for NAD^+^ reduction in *M. leprae* in vivo because, like *Mtb*, this pathogen can induce lipid droplet formation and utilize host lipids, specifically cholesterol. Several analogs synthesized in the current study (Figures 2A and S2) showed inhibition against mycobacterial 3β-Hsd at 50 μM, but only **16** (Figure S2) inhibited activity by greater than 50%. Compound **35** showed insignificant inhibitory activity against 3β-Hsd at 50 μM, indicating that its anti-TB action is likely not due to this activity.

After 3β-Hsd inhibition was excluded as the primary mechanism of action of **35**, we identified 6-azasteroid–protein interactions utilizing a nitrene analog of **35** (Diazirine PA, **40**) and another 6-azasteroid with a benzophenone side chain (Benzophenone PA, **44**, Figure 3B), both of which are capable of photo-cross-linking and which maintain potentiation activity in mycobacteria. Although our methodology captured a large number of proteins, as identified by mass spectrometry, we were able to select hit proteins by comparing data sets from biological replicates and multiple strategies for bead matrix enrichment. Filtering efforts were facilitated by robust CRISPR interference library data that have elucidated *Mtb* genes whose knockdown confers sensitivity to several anti-TB drugs. On the basis of the lack of activity of 6-azasteroids alone in *Mtb* or *Mm*, we reasoned that the vulnerability of protein targets or gene essentiality does not clearly correlate with azasteroid activity. 6-Azasteroids were active only in pairwise combinations with several individual anti-TB compounds; combinations with 6-azasteroids conferred enhanced mycobacterial sensitivity to each anti-TB compound. Therefore, we mined CRISPR interference data that uncovered drug sensitivity and not gene essentiality. Elucidating genes that are responsible for conferring sensitivity to approved anti-TB therapeutics is relevant for designing novel compounds, such as 6-azasteroids, that function by potentiating anti-TB therapies. Combining gene–drug sensitivity data with proteomic data enabled the identification of a small list of prioritized target proteins that interacted with 6-azasteroids and helped us to elucidate how 6-azasteroids may function. On the basis of validated functional annotations, the prioritized proteins provided strong evidence that one aspect of 6-azasteroid-mediated activity arises from the phenotypic impact of these compounds on ROS neutralization.

6-Azasteroids contribute to the sensitization of *Mm* and *Mtb* to ROS or limit the ability of these mycobacteria to neutralize oxidative stress (Figure 2B). Two proposed targets, Mtr and TrxB2, have been experimentally assessed to contribute to the thiol antioxidant–mediated detoxification of oxidative stress.^16,17^ One additional target, CydA, plays a role in maintaining the cellular proton motive force, which is required for ATP generation; and proton motive force uncoupling has been implicated in oxidative stress generation.^18,19^

Thioredoxin reductase (TrxB2 or TrxR) plays multiple roles in oxidative stress resistance and in essential processes such as the metabolism of sulfur and DNA. Thioredoxin reductases are highly conserved across bacteria and are required for growth in *Mtb* both in vitro and in mice. In the context of oxidative stress resistance, *trxB2* knockdown resulted in a small but statistically significant reduction in mycobacterial survival, as indicated by colony forming unit (CFU) counts in the presence of hydrogen peroxide. In MIC_99_ assays with hydrogen peroxide, only a 2-fold change is observed, indicating that the role of TrxB2 in detoxification is not as prominent as that of other proteins within the antioxidant system.^16^ These data correlate to results observed for treatment of *Mm* and *Mtb* with combinations of 6-azasteroids **2** or **35** and oxidants such as CHP (Figure S11).

Mycothiol reductase (Mtr) is responsible for reducing mycothione to mycothiol, an integral molecule in the antioxidant system of mycobacteria. The *mtr* gene has been shown to be actively transcribed during mycobacterial growth, and exposure to isoniazid, even at nonlethal doses, strongly upregulates *mtr* expression. In an *mtr* knockout *Mycobacterium smegmatis* strain, mycothiol levels were quantified by liquid chromatography–mass spectrometry.^20^ Interestingly, the levels were found to be the same in the *mtr* knockout as in the WT under standard aerobic conditions, but the intracellular mycothiol concentration decreased upon the addition of peroxide stress. Intracellular levels of mycothiol in *Mtb* are known to be in the millimolar range.^21^ Under standard conditions, the abundance of mycothiol may remain stable, but upon the introduction of oxidants, mycothiol represents a first-line strategy for detoxification. In the presence of *mtr* knockout, mycobacterial recycling of mycothiol is disabled, which leads to a decrease in the quantity of the reduced species. In *Corynebacterium glutamicum*, an actinobacterial species related to *M. smegmatis*, treatment of WT cultures with 10 mM CHP severely impacts survival, but this diminished-survival phenotype can be rescued when *mtr* is overexpressed by 5-fold. This reverse strategy, of using overexpression mutants rather than knockout or deletion mutants, elucidates the integral role that *mtr* plays in actinobacterial antioxidant systems.^17^

The function of these two antioxidant-mediating targets, TrxB2 and Mtr, and experimental data obtained with knockout, knockdown, or overexpression mutants support their potential as targets of 6-azasteroids (such as **35**) when analyzed in the context of phenotypic data obtained upon treatment of *Mm* and *Mtb* with 6-azasteroids. The role of mycothiol during antibiotic treatment supports the observed synergies between 6-azasteroids and BDQ. BDQ inhibits mycobacterial ATP synthesis within the electron transport chain, leading to respiratory poison via the generation of ROS. Considering that the key function of mycothiol is neutralization of oxidative stress, inhibition of Mtr and TrxB2 proteins, which mediate mycothiol levels, will prevent *Mtb* from responding appropriately to oxidation, resulting in synergy with ROS produced by BDQ treatment.^22^

*Mtb* has two terminal oxidases that contribute to aerobic respiration: cytochrome *bc*_1_*-aa*_3_ and cytochrome *bd* oxidase. The cyt *bc*_1_*-aa*_3_ supercomplex is energy efficient in that it pumps four protons; whereas cyt *bd* oxidase is less energetically efficient than cyt *bc*_1_*-aa*_3_; it aids in proton motive force generation by charge separation, but it does not pump protons.^23,24^ In vitro, CydA is a nonessential respiratory chain protein that constitutes one-half of the cyt *bd* oxidase heterodimer complex, the other half being CydB. CydA is the catalytically active component of cyt *bd* oxidase and binds electron-transporting quinols.^25^ Cyt *bd* oxidase is important for mycobacterial survival during infection because it maintains aerobic respiration under oxygen-limiting conditions, which are prevalent during pathogenesis.^26^ Deletion mutants of cyt *bd* oxidase show no growth defects under standard aerobic conditions. However, upon the addition of oxidative stress or antibiotics, mycobacterial killing is accentuated.^27,28^ In nonmycobacterial species, cyt *bd* oxidase has been shown to protect against oxidative stress under aerobic conditions based on the ability of bacteria to reduce dioxygen. In *M. thermoacetica*, cyt *bd* oxidase activity is required for neutralization of oxidative stress upon conversion from a hypoxic to a normoxic environment; this neutralization enables respiration.^29^ In *M. smegmatis*, peroxide treatment contributes to more significant killing of a cyt *bd* oxidase mutant than WT, resulting in nearly no loss of CFUs in WT and almost complete bactericidal effect in a cyt *bd* oxidase deletion mutant.^28^

When treated with BDQ, WT *Mtb* enters bacteriostasis by remodeling metabolism before BDQ exerts its bactericidal effects.^30^ In *M. smegmatis*, BDQ treatment results only in bacteriostasis and does not lead to cell death. However, in a cyt *bd* oxidase *M. smegmatis* deletion mutant, BDQ treatment has a bactericidal effect. Cyt *bd* oxidase–deficient *Mtb* strains treated with BDQ exhibit hypersensitivity in the form of more rapid kill kinetics (in that there is no longer a bacteriostasis period) and greater CFU reduction than in WT.^24^ Experiments in *M. smegmatis*, *Mtb*, and other bacteria highlight the impact of cyt *bd* oxidase in relation to bioenergetics-targeting antibiotics and oxidative stress protection. The phenotypic impact of 6-azasteroid treatment on *Mtb* and *Mm* under oxidative stress conditions, as well as in combination treatment with BDQ, mirrors the effects observed in a cyt *bd* oxidase deletion mutant and therefore provides evidence for a 6-azasteroid–CydA/cyt *bd* oxidase interaction.

Azasteroid activity increases under low-oxygen conditions, such that 6-azasteroids show bacteriostatic activity when used alone.^3^ These findings agree with several studies on differentially expressed genes that play a role in the ability of *Mtb* to persist during hypoxia. Rustad et al. observed increased expression of *trxB2* and *cydA* after 4 and 7 days of hypoxia, respectively.^31^ Analyses of an oxygen depletion time-course study by Peterson et al. showed that both *cydA* and *trxB2* are upregulated during mid and late hypoxia, respectively.^32^ The upregulation of these genes highlights the potential importance of the functions of their respective proteins, which enable *Mtb* to persist during the maintenance of the hypoxic response. Further experimentation will be required to experimentally validate the roles of these proteins in the context of the antimycobacterial activity of 6-azasteroids both in vitro and in vivo.

Previously, several Mce3R-regulated genes within the *mel2* operon, genes that are reported to contribute to oxidative stress resistance, were implicated in the potentiation activity of 6-azasteroids.^33^ Interestingly, none of the protein products of these genes were identified as prioritized hits for 6-azasteroid–probe interactions in the current study.

Sequencing of 6-azasteroid-resistant mutants did not identify *mce3R* itself, Mce3R-regulated genes, or related genes, instead identifying mutations in *PE*/*PPE* genes,^6^ which are highly variable in *Mtb*. Although proteins that are regulated by Mce3R were not detected in interaction assays, we have previously shown that these proteins play a supplementary role in mediating antibiotic stress under 6-azasteroid treatment.^6^ Results from this study indicate that the mechanisms of *mel2*-mediated azasteroid activity may be indirect and not a consequence of 6-azasteroid–Mel2 binding. Further experimentation will be necessary to fully elucidate the role of Mce3R-regulated proteins in mediating 6-azasteroid activity.

As demonstrated in the work described herein, whole-cell-based phenotypic screens require subsequent work to uncover how molecules interact with the pathogen to generate an antibacterial effect.^5^ These mechanistic studies are required for optimizing and advancing lead compounds for further development. The difficulty of elucidating the drug mechanism of action represents a major bottleneck in the phenotypic-screening approach because drug–target interactions can be complex. As indicated by our current investigation into the mechanism of action of 6-azasteroids, including the identification of several enriched proteins in interaction assays, our previous discovery that Mce3R-regulated genes are required for 6-azasteroid activity, and our prior inability to isolate single-gene resistance mutants, the sensitization of mycobacteria to antibiotics by 6-azasteroids is likely due to complex polypharmacology in oxidative stress-related systems.

## Methods

### Synthesis of 6-Azasteroid Probes

6-Azasteroid inhibitors were synthesized as previously described.^7,34^ The diazirine linker [2-(3-butyn-1-yl)-3*H*-diaziren-3-yl]acetic acid (CAS No. 2049109-24-0) was purchased from AmBeed.^35^

#### 17β-[*N*-(4-Trifluoromethoxy-diphenylmethyl)carbamoyl]-6-propyl-azaandrostan-3-[2-(3-but-3-ynyldiazirin-3-yl) acetate] (**40**, Diazirine PA)

17β-[*N*-(4-Trifluoromethoxy-diphenylmethyl)carbamoyl]-6-propyl-azaandrostan-3-one (**35**, 50 mg, 1 equiv) was placed in a round-bottom flask along with 5 mL of anhydrous methanol, and the resulting solution was stirred at room temperature. Then NaBH_4_ (1.1 equiv) was added slowly, and the reaction was allowed to proceed for at least 2 h or until completion, with monitoring by TLC. Excess ethyl acetate was added to quench the reaction, and the solvent was removed in vacuo. The resulting 3-hydroxy-6-azasteroid was dissolved in DCM, the solution was washed with water, and the aqueous phase was extracted with 3 × 10 mL of DCM. The DCM extracts containing the reduced 3-hydroxy-6-azasteroid were dried with anhydrous MgSO_4_, and the solvent was removed in vacuo.

The diazirine linker [2-(3-butyn-1-yl)-3*H*-diaziren-3-yl]acetic acid was dissolved in anhydrous DCM, and the solution was cooled to 0 °C in an ice–water bath. To the linker suspension were added 1-ethyl-3-(3-(dimethylamino)propyl)carbodiimide (1.2 equiv) and 10% dimethylaminopyridine (DMAP, 0.12 equiv), and the reaction mixture was stirred for 1–2 h. Then the 3-hydroxy-6-azasteroid intermediate (2 equiv) was added, and the resulting mixture was allowed to come to room temperature and stir overnight. The progress of the reaction was monitored by TLC. Upon completion, the reaction mixture was washed with excess water to remove urea byproduct, and the organic phase was dried over anhydrous MgSO_4_ and filtered. The solvent was removed from the filtrate by rotary evaporation, and the residue was purified by flash chromatography (0–20% methanol in DCM) to afford **40** (Figure S12A).

#### 17β-[*N*-[4-(4-Aminobenzoyl)phenyl]pent-4-ynamide]-6-azaandrost-4-en-3-one (**44**, Benzophenone PA)

Pent-4-ynoic acid (Alfa Aesar, CAS 6089-09-4) was dissolved in anhydrous toluene (5 mL). Pyridine (2.6 equiv, Sigma-Aldrich) and one drop of DMF (∼5 μL, Sigma-Aldrich) were added, and the mixture was stirred over an ice–water bath for 5 min. Thionyl chloride (1.7 equiv, Alfa Aesar) was added in a dropwise fashion. After the mixture was stirred for 1–2 h at 0 °C, toluene was removed by rotary evaporation, and the resulting acid chloride intermediate was resuspended in anhydrous DCM. A solution of (4,4′-diaminobenzophenone (AmBeed, CAS No. 611-98-3) in anhydrous DMF containing TEA (1.5 equiv) was added dropwise to the acid chloride intermediate over an ice–water bath. The resulting mixture was stirred for 1 h at 0 °C and then at room temperature overnight with monitoring by TLC. Upon completion of the reaction, the solvent was removed in vacuo, and the residue was purified by flash chromatography to afford *N*-(4-(4′-aminobenzoyl)phenyl)pent-4-ynamide.

17β-[6-*tert*-Butyloxycarbonyl-azaandrost-4-en-3-one]carboxylic acid was suspended in anhydrous toluene (5 mL). Pyridine (2.6 equiv, Sigma-Aldrich) and one drop of DMF (∼5 μL, Sigma-Aldrich) were added, and the mixture was stirred over an ice–water bath for 5 min. Thionyl chloride (1.7 equiv, Alfa Aesar) was added in a dropwise fashion, and the reaction mixture was stirred for 1–2 h at 0 °C over an ice–water bath. Toluene was removed by rotary evaporation, and the intermediate acid chloride was resuspended in anhydrous DCM. A solution of *N*-(4-(4′-aminobenzoyl)phenyl)pent-4-ynamide in anhydrous DMF containing TEA (1.5 equiv) was added dropwise to the acid chloride over an ice–water bath. The resulting mixture was stirred for 1 h at 0 °C and then at room temperature overnight with monitoring by TLC. Upon completion of the reaction, the solvent was removed in vacuo and the residue was purified by flash chromatography. To a DCM solution of the resulting 17β-[*N*-[4-(4-aminobenzoyl)phenyl]pent-4-ynamide]-6-*tert*-butyloxycarbonyl-azaandrost-4-en-3-one was added 1–2 mL of TFA. The reaction was stirred at room temperature for 2 h with monitoring by TLC. Upon completion of the reaction, the TFA was quenched with saturated aqueous NaHCO_3_. The aqueous phase was extracted with 3 × 10 mL of DCM and dried over anhydrous MgSO_4_, and the solvent was removed in vacuo to afford **44** (Figure S12A).

Compounds **40** and **44** were assessed to be greater than 95% pure by ^1^H NMR spectroscopy.

### Bacterial Strains and Culturing

Mycobacterial cultures were grown in poly(ethylene terephthalate glycol) Erlenmeyer flasks of varying sizes (Grenier Bio-One). Both *Mtb (*CDC1551) and *Mm* (BAA-535) cultures were grown in Middlebrook 7H9 (g/L: 2.5 g of disodium phosphate, 1.0 g of monopotassium phosphate, 0.5 g of monosodium glutamate, 0.5 g of ammonium sulfate, 0.1 g of sodium citrate, 0.05 g of magnesium sulfate, 0.04 g of ferric ammonium citrate, 1.0 mg of copper sulfate, 1.0 mg of pyridoxine HCl, 1.0 mg of zinc sulfate, 0.5 mg of biotin, 0.5 mg of calcium chloride; pH 6.6 at 25 °C).

### MIC_99_ Assays of 6-Azasteroids

6-Azasteroids were assayed for their ability to potentiate the inhibition of mycobacterial growth upon treatment with BDQ. In non-tissue-culture-treated 96-well plates, the 6-azasteroids were held at a fixed concentration of 20 μM, whereas BDQ was added to wells by serial dilution in Middlebrook 7H9 media. *Mtb (*CDC1551) or *Mm* (BAA-535) was cultured in 7H9 supplemented with glycerol and diluted to an OD_600_ of 0.02. Diluted mycobacterium culture (50 μL) was added to the combined drug solutions (50 μL) in the wells, and the plates were incubated at 36 °C for 2 weeks (*Mtb*) or at 30 °C for 5 days (*Mm*). Mycobacterial growth inhibition was measured by Alamar Blue staining (*Mm*) or by visual inspection (*Mtb*)*.* Results were plotted in a dose–response curve against the log of the BDQ concentration and fitted with a nonlinear Gompertz curve. MIC_99_ values are reported as micromolar drug concentrations.

### Cytotoxicity Assay

HepG2 or THP-1 cells were utilized to assess the cytotoxicity of 6-azasteroids using the method outlined by Miret et al.^36^ Cells were purchased from ATCC (cat. nos. HB-8065 and TIB-202) were cultured from frozen stock grown on Minimum Eagle Medium (Corning) or Roswell Park Memorial Institute 1640 medium (Corning) supplemented with 10% fetal bovine serum and 0.1% penicillin/streptomycin. Cells were plated in 96-well plates at 10^4^ cells/well. Azasteroids were dissolved in DMSO to yield a 20 mM stock solution, and serial dilutions were prepared. Assays were performed in technical triplicates. 6-Azasteroids were incubated with cells for 72 h, after which Alamar Blue staining was performed. Data were analyzed by determining the concentration of the oxidized Alamar Blue in each well. Results were plotted in a dose–response curve against the log of 6-azasteroid concentration and were fitted with a nonlinear fit curve. IC_50_ values are reported as micromolar drug concentrations.

### Assessment of 3β-Hsd Inhibition

Recombinant *Mtb* 3β-Hsd (Rv1106c) was cloned from genomic DNA and expressed and purified as previously described.^10^ The activity of the recombinant protein was assayed at 25 °C for the linear portion of the reaction (<10% conversion) by monitoring the absorbance of NADH at 340 nm. 3β-Hsd was diluted with [tris(hydroxymethyl)methylamino]propanesulfonic acid buffer (100 mM, pH 8.5) to a final concentration of 0.5 μM, and the buffer was supplemented with 30 mM MgCl_2_ and 150 mM NaCl. A 10 mM stock solution of NAD^+^ cofactor was diluted to a final concentration of 350 μM, which is 1.75 times the reported *K*_m_ (200 μM). A 3 mM stock solution of dehydroepiandrosterone was prepared in ethanol. For each reaction, the final volume of ethanol was kept constant at 5%; the final concentration was 220 μM, which is 1.83 times the reported *K*_m_ (120 μM). 6-Azasteroids reconstituted with DMSO were equilibrated with 3β-Hsd, and the reactions were initiated by the addition of NAD^+^. Reactions were carried out in quartz cuvettes and monitored at 340 nm.

### ROS Sensitivity

Exponentially growing *Mtb* WT CDC1551 or *Mm* WT BAA-535 strains cultured in 7H9 medium supplemented with 0.4% glycerol or propionate were treated with 6-azasteroid for 6 h prior to exposure to CHP for 30 min. Cultures were treated with CellROX Green (Life Technologies) at a final concentration of 5 μM for 30 min at 37 °C. The cells were pelleted, the supernatant was discarded, and the pelleted cells were washed with 7H9 medium to remove any extracellular CellROX Green. The washed cells were resuspended in phosphate buffered saline and analyzed on a fluorescence plate reader at excitation/emission wavelengths of 485/565 nm.

### Measurement of ATP Concentration

*Mtb* or *Mm* in the exponential phase of growth was exposed to 0.4, 0.1, 0.025, or 0 μM BDQ in combination with 20, 5, or 0 μM **2** or **35** for 96 h. Aliquots (1.5 mL) of bacterial suspension were removed and mixed with 3 mL of boiling Tris-EDTA reagent (100 mM Tris, 4 mM EDTA, pH 7.75); and cells were lysed for 2 min with glass beads, heated at 100 °C for 5 min, and cooled on ice. Cell debris was removed by centrifugation. Supernatants were collected; an equal volume of luciferase reagent (ATP Bioluminescence Assay Kit HS II, Roche) was added to the supernatants; and luminescence was measured. ATP was measured with an ATP Colorimetric/Fluorometric Assay Kit (BioVision Research Products) according to the manufacturer’s protocol. The survival of mycobacteria was measured by plating them on 7H11 agar plates and counting CFUs to normalize the ATP concentration to the number of live mycobacterial cells.

### Protein Target Identification by Affinity Capture

*Mm* was cultured on 7H9 media (100 mL) supplemented with glycerol (2 mL/L) or cholesterol (0.5 mM); grown to OD_600_ = 1; and concentrated 10-fold by centrifugation. The concentrated culture was treated for 6 h at 30 °C with 50 μM of a probe (**40** or **44**), either alone or with a 10-fold excess of the nonprobe analog (**43**). Treated cultures were irradiated over an ice–water bath with a 100-W UV lamp (Spectroline SB100P, rated 4800 μW/cm^2^ at 38 cm), followed by bead beating lysis in the presence of detergent (10% v/v NP-40 and 5% w/v sodium deoxycholate). Lysates were depleted of endogenous biotin and biotin-binding proteins by incubation with streptavidin–agarose for 30 min. Protein concentration was quantified by means of a bicinchonic acid assay^37^ and adjusted to 1.0–2.0 mg/mL with phosphate buffered saline. The UV-cross-linked lysates were then subjected to click reaction: azido-PEG3-TAMRA-biotin (100 μM) or azide agarose beads or azido-functionalized magnetic nanoparticles, sodium d-ascorbate (2.5 mM), copper(II) sulfate pentahydrate (1 mM), and tris(benzyltriazolylmethyl)amine (2 mM). Streptavidin bead enrichment was carried out by means of click reactions with azido-PEG3-TAMRA-biotin, and full-length proteins captured via biotin–streptavidin interaction were eluted from the beads by boiling them in 2× Laemmli buffer. Captured proteins were analyzed via SDS-PAGE and then by antistreptavidin Western blot. Western blotting was performed by blocking with 0.22-μm-filtered 3% bovine serum albumin followed by streptavidin-conjugated horseradish peroxidase (Genscript, cat. no. M00091) incubation overnight at 1:1000 dilution in Tris-buffered saline containing 0.1% Tween 20. For proteomic analysis, azide agarose beads or azido-functionalized magnetic nanoparticles were thoroughly washed with 6 M urea and phosphate buffered saline, and the protein-bearing beads or nanoparticles were subjected to on-bead trypsin digestion in 50 mM tetraethylammonium bromide buffer (pH 8) overnight at 37 °C.

### Proteomic Analysis

The resulting peptides were dried under a vacuum and resuspended in 0.1% formic acid. Peptides were analyzed by C18 reverse-phase liquid chromatography–tandem mass spectrometry. HPLC C18 columns were prepared using a P-2000 CO_2_ laser puller (Sutter Instruments) and silica tubing (100 μm inner diameter × 20 cm) and were self-packed with 3-μm Reprosil resin. Peptides typically were separated at a flow rate of 300 nL/min with a gradient elution step changing from 0.1% formic acid to 40% acetonitrile over 90 min followed by 90% acetonitrile wash and re-equilibration steps. Parent peptide masses and collision-induced fragment masses were collected using an orbital trap instrument (Q-Exactive HF, Thermo), and protein databases were searched with Proteome Discoverer software (version 2.4, ThermoFisher Scientific). Electrospray ionization was achieved at a spray voltage of ∼2.3 kV. Information-dependent mass spectrometry and tandem mass spectrometry acquisitions were made using a 100 ms survey scan (*m*/*z* 375–1400) at 60,000 resolution followed typically by “top 20” consecutive second product ion scans at 15,000 resolution. False discovery rates for protein levels and spectra were set to 0.01 (1%) cutoffs. Proteome Discoverer (ver. 2.4) was used for data analysis of peptide sequences that were annotated to the proteome of *Mm* strain ATCC BAA-535 (Proteome ID: UP000001190).

### *In Silico* Molecular Docking

AutoDock Vina^38^ was utilized for molecular receptor–ligand docking of 6-azasteroids with X-ray crystallographic structures or AlphaFold-modeled structures of the hit proteins identified in proteomics experiments. Two-dimensional inhibitor structures were developed using ChemDraw (ver. 19.1) software and were exported as mol files for use with visualization software. The UCSF Chimera extensible molecular modeling system was used as the visualization software for AutoDock Vina. The Autodock Tools plugin was used to prepare the ligand and receptor for docking operations, including to convert inhibitors to three-dimensional structures. The search box was situated over the active site of each protein in various sizes. Active sites were predicted by the PrankWeb server;^34^ and the top 10 binding poses were predicted.

## Data Availability

Proteomics data sets from this study are deposited in the MassIVE database under reference MSV000091952.

## Abbreviations

ATP: adenine triphosphate
BDQ: bedaquiline
Benzophenone PA: 17β-[*N*-[4-(4-aminobenzoyl)phenyl]pent-4-ynamide]-6-azaandrost-4-en-3-one
CFUs: colony forming units
CHP: cumene hydroperoxide
CRISPR interference: clustered regularly interspaced short palindromic repeats interference
CuAAC: copper-catalyzed azide–alkyne cycloaddition
DCM: dichloromethane
Diazirine PA: 17β-[*N*-(4-trifluoromethoxy-diphenylmethyl)carbamoyl]-6-propyl-azaandrost-4-en-3-[2-(3-but-3-ynyldiazirin-3-yl) acetate]
DMF: *N*,*N*-dimethylformamide
DMSO: dimethyl sulfoxide
EDC: 1-ethyl-3-(3-(dimethylamino)propyl)carbodiimide
HPLC: high-performance liquid chromatography
Hsd: hydroxysteroid dehydrogenase
IC_50_: inhibitory concentration required to inhibit 50% activity/growth
kDa: kilodaltons
KEGG: Kyoto Encyclopedia of Genes and Genomes
MDR-TB: multidrug-resistant tuberculosis
MK9: menaquinone-9
MIC_99_: minimal inhibitory concentration
*Mm*: Mycobacterium *marinum*
*Mtb*: Mycobacterium *tuberculosis*
NAD^+^: nicotinamide adenine dinucleotide (oxidized form)
NADH: nicotinamide adenine dinucleotide (reduced form)
NADP^+^: nicotinamide adenine dinucleotide phosphate (oxidized form)
NT: no treatment
OD_600_: optical density at 600 nm
PEG: poly(ethylene glycol)
RFU: relative fluorescence units
ROS: reactive oxygen species
SDS-PAGE: sodium dodecyl sulfate polyacrylamide gel electrophoresis
TAMRA: carboxytetramethylrhoadamine fluorophore
TB: tuberculosis
TEA: triethylamine
TFA: trifluoroacetic acid
TLC: thin-layer chromatography
UV: ultraviolet
WT: wild type

## References

[ref1] Global Tuberculosis Report 2022; WHO, 2021; p 23.

[ref2] Yuan T.; Sampson N. S. Hit Generation in TB Drug Discovery: From Genome to Granuloma. Chem. Rev. 2018, 118 (4), 1887–1916. 10.1021/acs.chemrev.7b00602. 29384369 PMC5832989

[ref3] Palomino J. C.; Martin A. Drug Resistance Mechanisms in *Mycobacterium tuberculosis*. Antibiotics (Basel) 2014, 3 (3), 317–40. 10.3390/antibiotics3030317. 27025748 PMC4790366

[ref4] Torella J. P.; Chait R.; Kishony R. Optimal Drug Synergy in Antimicrobial Treatments. PLoS Comput. Biol. 2010, 6 (6), e1000796 10.1371/journal.pcbi.1000796. 20532210 PMC2880566

[ref5] Yuan T.; Werman J. M.; Sampson N. S. The Pursuit of Mechanism of Action: Uncovering Drug Complexity in TB Drug Discovery. RSC Chem. Biol. 2021, 2 (2), 423–440. 10.1039/D0CB00226G. 33928253 PMC8081351

[ref6] Yang X.; Yuan T.; Ma R.; Chacko K. I.; Smith M.; Deikus G.; Sebra R.; Kasarskis A.; van Bakel H.; Franzblau S. G.; Sampson N. S. Mce3R Stress-Resistance Pathway Is Vulnerable to Small-Molecule Targeting That Improves Tuberculosis Drug Activities. ACS Infect Dis 2019, 5 (7), 1239–1251. 10.1021/acsinfecdis.9b00099. 31012313 PMC6630528

[ref7] Frye S. V.; Haffner C. D.; Maloney P. R.; Mook R. A. Jr.; Dorsey G. F. Jr.; Hiner R. N.; Cribbs C. M.; Wheeler T. N.; Ray J. A.; Andrews R. C.; Batchelor K. W.; Bramson H. N.; Stuart J. D.; Schweiker S. L.; van Arnold J.; Croom S.; Bickett D. M.; Moss M. L.; Tian G.; Unwalla R. J.; Lee F. W.; Tippin T. K.; James M. K.; Grizzle M. K.; Long J. E.; Schuster S. V. 6-Azasteroids: Structure-Activity Relationships for Inhibition of type 1 and 2 Human 5 alpha-Reductase and Human Adrenal 3 beta-Hydroxy-delta 5-Steroid Dehydrogenase/3-Keto-delta 5-Steroid Isomerase. J. Med. Chem. 1994, 37 (15), 2352–2360. 10.1002/chin.199451227. 8057283

[ref8] Thomas S. T.; Yang X.; Sampson N. S. Inhibition of the *M. tuberculosis* 3beta-Hydroxysteroid Dehydrogenase by Azasteroids. Bioorg. Med. Chem. Lett. 2011, 21 (8), 2216–9. 10.1016/j.bmcl.2011.03.004. 21439822 PMC3077731

[ref9] Rosa T.; Marques M. A. M.; DeBoard Z.; Hutchins K.; Silva C. A. A.; Montague C. R.; Yuan T.; Amaral J. J.; Atella G. C.; Rosa P. S.; Mattos K. A.; VanderVen B. C.; Lahiri R.; Sampson N. S.; Brennan P. J.; Belisle J. T.; Pessolani M. C. V.; Berredo-Pinho M. Reductive Power Generated by *Mycobacterium leprae* through Cholesterol Oxidation Contributes to Lipid and ATP Synthesis. Front Cell Infect Microbiol 2021, 11, 709972 10.3389/fcimb.2021.709972. 34395315 PMC8355898

[ref10] Yang X.; Dubnau E.; Smith I.; Sampson N. S. Rv1106c from *Mycobacterium tuberculosis* Is a 3beta-Hydroxysteroid Dehydrogenase. Biochemistry 2007, 46 (31), 9058–67. 10.1021/bi700688x. 17630785 PMC2596615

[ref11] Duckworth B. P.; Wilson D. J.; Nelson K. M.; Boshoff H. I.; Barry C. E. 3rd; Aldrich C. C. Development of a Selective Activity-Based Probe for Adenylating Enzymes: Profiling MbtA Involved in Siderophore Biosynthesis from *Mycobacterium tuberculosis*. ACS Chem. Biol. 2012, 7 (10), 1653–8. 10.1021/cb300112x. 22796950 PMC3477287

[ref12] Li S.; Poulton N. C.; Chang J. S.; Azadian Z. A.; DeJesus M. A.; Ruecker N.; Zimmerman M. D.; Eckartt K. A.; Bosch B.; Engelhart C. A.; Sullivan D. F.; Gengenbacher M.; Dartois V. A.; Schnappinger D.; Rock J. M. Crispri Chemical Genetics and Comparative Genomics Identify Genes Mediating Drug Potency in *Mycobacterium tuberculosis*. Nat. Microbiol 2022, 7 (6), 766–779. 10.1038/s41564-022-01130-y. 35637331 PMC9159947

[ref13] Krivak R.; Hoksza D. P2rank: Machine Learning Based Tool for Rapid and Accurate Prediction of Ligand Binding Sites from Protein Structure. J. Cheminform. 2018, 10 (1), 39 10.1186/s13321-018-0285-8. 30109435 PMC6091426

[ref14] Jumper J.; Evans R.; Pritzel A.; Green T.; Figurnov M.; Ronneberger O.; Tunyasuvunakool K.; Bates R.; Zidek A.; Potapenko A.; Bridgland A.; Meyer C.; Kohl S. A. A.; Ballard A. J.; Cowie A.; Romera-Paredes B.; Nikolov S.; Jain R.; Adler J.; Back T.; Petersen S.; Reiman D.; Clancy E.; Zielinski M.; Steinegger M.; Pacholska M.; Berghammer T.; Bodenstein S.; Silver D.; Vinyals O.; Senior A. W.; Kavukcuoglu K.; Kohli P.; Hassabis D. Highly Accurate Protein Structure Prediction with Alphafold. Nature 2021, 596 (7873), 583–589. 10.1038/s41586-021-03819-2. 34265844 PMC8371605

[ref15] Jeffreys L. N.; Ardrey A.; Hafiz T. A.; Dyer L. A.; Warman A. J.; Mosallam N.; Nixon G. L.; Fisher N. E.; Hong W. D.; Leung S. C.; Aljayyoussi G.; Bibby J.; Almeida D. V.; Converse P. J.; Fotouhi N.; Berry N. G.; Nuermberger E. L.; Upton A. M.; O’Neill P. M.; Ward S. A.; Biagini G. A. Identification of 2-Aryl-Quinolone Inhibitors of Cytochrome Bd and Chemical Validation of Combination Strategies for Respiratory Inhibitors against *Mycobacterium tuberculosis*. ACS Infect Dis 2023, 9 (2), 221–238. 10.1021/acsinfecdis.2c00283. 36606559 PMC9926492

[ref16] Lin K.; O’Brien K. M.; Trujillo C.; Wang R.; Wallach J. B.; Schnappinger D.; Ehrt S. *Mycobacterium tuberculosis* Thioredoxin Reductase Is Essential for Thiol Redox Homeostasis but Plays a Minor Role in Antioxidant Defense. PLoS Pathog 2016, 12 (6), e1005675 10.1371/journal.ppat.1005675. 27249779 PMC4889078

[ref17] Si M.; Zhao C.; Zhang B.; Wei D.; Chen K.; Yang X.; Xiao H.; Shen X. Overexpression of Mycothiol Disulfide Reductase Enhances *Corynebacterium glutamicum* Robustness by Modulating Cellular Redox Homeostasis and Antioxidant Proteins under Oxidative Stress. Sci. Rep. 2016, 6, 29491 10.1038/srep29491. 27383057 PMC4935862

[ref18] Rao S. P.; Alonso S.; Rand L.; Dick T.; Pethe K. The Protonmotive Force Is Required for Maintaining Atp Homeostasis and Viability of Hypoxic, Nonreplicating *Mycobacterium tuberculosis*. Proc. Natl. Acad. Sci. U. S. A. 2008, 105 (33), 11945–50. 10.1073/pnas.0711697105. 18697942 PMC2575262

[ref19] Mascolo L.; Bald D. Cytochrome Bd in Mycobacterium Tuberculosis: A Respiratory Chain Protein Involved in the Defense against Antibacterials. Prog. Biophys. Mol. Biol. 2020, 152, 55–63. 10.1016/j.pbiomolbio.2019.11.002. 31738981

[ref20] Holsclaw C. M.; Muse W. B. 3rd; Carroll K. S.; Leary J. A. Mass Spectrometric Analysis of Mycothiol Levels in Wild-Type and Mycothiol Disulfide Reductase Mutant *Mycobacterium smegmatis*. Int. J. Mass Spectrom. 2011, 305 (2–3), 151–156. 10.1016/j.ijms.2010.10.027. 21857792 PMC3156591

[ref21] Bhaskar A.; Chawla M.; Mehta M.; Parikh P.; Chandra P.; Bhave D.; Kumar D.; Carroll K. S.; Singh A. Reengineering Redox Sensitive GFP to Measure Mycothiol Redox Potential of *Mycobacterium tuberculosis* During Infection. PLoS Pathog 2014, 10 (1), e1003902 10.1371/journal.ppat.1003902. 24497832 PMC3907381

[ref22] Torfs E.; Piller T.; Cos P.; Cappoen D. Opportunities for Overcoming *Mycobacterium tuberculosis* Drug Resistance: Emerging Mycobacterial Targets and Host-Directed Therapy. Int. J. Mol. Sci. 2019, 20 (12), 2868 10.3390/ijms20122868. 31212777 PMC6627145

[ref23] Bajeli S.; Baid N.; Kaur M.; Pawar G. P.; Chaudhari V. D.; Kumar A. Terminal Respiratory Oxidases: A Targetable Vulnerability of Mycobacterial Bioenergetics?. Front Cell Infect Microbiol 2020, 10, 589318 10.3389/fcimb.2020.589318. 33330134 PMC7719681

[ref24] Borisov V. B.; Gennis R. B.; Hemp J.; Verkhovsky M. I. The Cytochrome BD Respiratory Oxygen Reductases. Biochim. Biophys. Acta 2011, 1807 (11), 1398–413. 10.1016/j.bbabio.2011.06.016. 21756872 PMC3171616

[ref25] Safarian S.; Opel-Reading H. K.; Wu D.; Mehdipour A. R.; Hards K.; Harold L. K.; Radloff M.; Stewart I.; Welsch S.; Hummer G.; Cook G. M.; Krause K. L.; Michel H. The Cryo-Em Structure of the Bd Oxidase from *M. tuberculosis* Reveals a Unique Structural Framework and Enables Rational Drug Design to Combat TB. Nat. Commun. 2021, 12 (1), 5236 10.1038/s41467-021-25537-z. 34475399 PMC8413341

[ref26] Shi L.; Sohaskey C. D.; Kana B. D.; Dawes S.; North R. J.; Mizrahi V.; Gennaro M. L. Changes in Energy Metabolism of *Mycobacterium tuberculosis* in Mouse Lung and under *in vitro* Conditions Affecting Aerobic Respiration. Proc. Natl. Acad. Sci. U. S. A. 2005, 102 (43), 15629–34. 10.1073/pnas.0507850102. 16227431 PMC1255738

[ref27] Berney M.; Hartman T. E.; Jacobs W. R. Jr. A *Mycobacterium tuberculosis* Cytochrome BD Oxidase Mutant Is Hypersensitive to Bedaquiline. mBio 2014, 5 (4), e01275-14 10.1128/mBio.01275-14. 25028424 PMC4161257

[ref28] Lu P.; Heineke M. H.; Koul A.; Andries K.; Cook G. M.; Lill H.; van Spanning R.; Bald D. The Cytochrome Bd-Type Quinol Oxidase Is Important for Survival of *Mycobacterium smegmatis* under Peroxide and Antibiotic-Induced Stress. Sci. Rep 2015, 5, 10333 10.1038/srep10333. 26015371 PMC4450806

[ref29] Das A.; Silaghi-Dumitrescu R.; Ljungdahl L. G.; Kurtz D. M. Jr. Cytochrome BD Oxidase, Oxidative Stress, and Dioxygen Tolerance of the Strictly Anaerobic Bacterium Moorella Thermoacetica. J. Bacteriol. 2005, 187 (6), 2020–9. 10.1128/JB.187.6.2020-2029.2005. 15743950 PMC1064043

[ref30] Koul A.; Vranckx L.; Dhar N.; Gohlmann H. W.; Ozdemir E.; Neefs J. M.; Schulz M.; Lu P.; Mortz E.; McKinney J. D.; Andries K.; Bald D. Delayed Bactericidal Response of *Mycobacterium tuberculosis* to Bedaquiline Involves Remodelling of Bacterial Metabolism. Nat. Commun. 2014, 5, 3369 10.1038/ncomms4369. 24569628 PMC3948051

[ref31] Rustad T. R.; Harrell M. I.; Liao R.; Sherman D. R. The Enduring Hypoxic Response of *Mycobacterium tuberculosis*. PLoS One 2008, 3 (1), e1502 10.1371/journal.pone.0001502. 18231589 PMC2198943

[ref32] Peterson E. J. R.; Abidi A. A.; Arrieta-Ortiz M. L.; Aguilar B.; Yurkovich J. T.; Kaur A.; Pan M.; Srinivas V.; Shmulevich I.; Baliga N. S. Intricate Genetic Programs Controlling Dormancy in *Mycobacterium tuberculosis*. Cell Rep 2020, 31 (4), 107577 10.1016/j.celrep.2020.107577. 32348771 PMC7605849

[ref33] Subbian S.; Mehta P. K.; Cirillo S. L.; Cirillo J. D. The *Mycobacterium marinum mel2* Locus Displays Similarity to Bacterial Bioluminescence Systems and Plays a Role in Defense against Reactive Oxygen and Nitrogen Species. BMC Microbiol. 2007, 7, 4 10.1186/1471-2180-7-4. 17239244 PMC1793995

[ref34] Haffner C. Synthesis of 6-Azacholesten-3-ones: Potent Inhibitors of 5α-Reductase. Tetrahedron Lett. 1995, 36 (23), 4039–4042. 10.1016/0040-4039(95)00711-K.

[ref35] Li Z.; Hao P.; Li L.; Tan C. Y.; Cheng X.; Chen G. Y.; Sze S. K.; Shen H. M.; Yao S. Q. Design and Synthesis of Minimalist Terminal Alkyne-Containing Diazirine Photo-Crosslinkers and Their Incorporation into Kinase Inhibitors for Cell- and Tissue-Based Proteome Profiling. Angew. Chem., Int. Ed. Engl. 2013, 52 (33), 8551–6. 10.1002/anie.201300683. 23754342

[ref36] Miret S.; De Groene E. M.; Klaffke W. Comparison of in Vitro Assays of Cellular Toxicity in the Human Hepatic Cell Line HepG2. J. Biomol Screen 2006, 11 (2), 184–93. 10.1177/1087057105283787. 16314402

[ref37] Smith P. K.; Krohn R. I.; Hermanson G. T.; Mallia A. K.; Gartner F. H.; Provenzano M. D.; Fujimoto E. K.; Goeke N. M.; Olson B. J.; Klenk D. C. Measurement of Protein Using Bicinchoninic Acid. Anal. Biochem. 1985, 150 (1), 76–85. 10.1016/0003-2697(85)90442-7. 3843705

[ref38] Jendele L.; Krivak R.; Skoda P.; Novotny M.; Hoksza D. Prankweb: A Web Server for Ligand Binding Site Prediction and Visualization. Nucleic Acids Res. 2019, 47 (W1), W345–W349. 10.1093/nar/gkz424. 31114880 PMC6602436

