# Supplementary material for: A Chemoproteomic Approach to Elucidate the Mechanism of Action of 6-Azasteroids with Unique Activity in Mycobacteria
Source: ACS Infect Dis. 2023 Sep 29;9(10):1993–2004. doi: 10.1021/acsinfecdis.3c00296 (PMC10580313; doi:10.1021/acsinfecdis.3c00296)
Supplement: Supplementary file 1 — id3c00296_si_001.pdf [file id3c00296_si_001.pdf]

Supplementary Materials for

A Chemoproteomic Approach to Elucidate the Mechanism of Action of 6-Azasteroids with Unique Activity in Mycobacteria

Joshua M. Werman,[a] Yu-Ching Chen,[b] Tianao Yuan,[a] Xinxin Yang,[a] Nicole S. Sampson[a,c,*]

[a]Department of Chemistry, Stony Brook University, Stony Brook, NY 11794-3400
[b]Program in Biochemistry and Structural Biology, Stony Brook University, Stony Brook, NY 11794-5215
[c]Department of Chemistry, University of Rochester, Rochester, NY 14627-0216

*Corresponding author:
nicole.sampson@rochester.edu

Table of Contents

Table S1. Overview of 6-azasteroid modifications. 3

Table S2. Overview of 6-azasteroids – activity & cytotoxicity. 5

Figure S1. Synthesis of 6-azasteroids. 14

Figure S2. Structures of 6-azasteroids tested for inhibition of Mtb 3β-hydroxysteroid dehydrogenase. 15

Figure S3. (A) Full western blot & (B) full fluorescence image of whole cell lysate interaction assay, proteins enriched via azido-PEG3-TAMRA-biotin. 16

Figure S4. KEGG ID functional analysis of the entire Mm proteome (A) and the entire proteomics data set (B) by top 20 functional categories. 19

Figure S5. Enrichment methods comparison by Venn diagram 20

Figure S6. CRISPRi negative screen of selected genes. 24

Figure S7. Overview of pull-down assay data analysis. 25

Figure S8. Docking of azasteroids to CydA (Rv1623c). 26

Figure S9. Docking of azasteroids to Mtr (Rv2855). 27

Figure S10. Docking of azasteroids to TrxB2 (Rv3913). 28

Figure S11. Survival of Mtb cultures treated with peroxide alone and in combination with 6-azasteroid. 29

Figure S12. Synthesis of 6-azasteroid probes. 30

Figure S13. Nuclear magnetic resonance of compounds 40 and 44. 32

Reference. 33

Table S1. Overview of 6-azasteroid modifications.

Com-pound No.	R_1	R_2	C_4-C_5
**1**	(2, 5-di-*t*-butyl)anilide	H	alkene
**2**	(2, 5-di-*t*-butyl)anilide	propyl	alkene
**3**	(2, 5-di-*t*-butyl)anilide	H	alkyl
**4**	(2, 5-di-*t*-butyl)anilide	propyl	alkyl
**5**	(2-*t*-butyl, 5-trifluoromethyl)anilide	H	alkene
**6**	(2-*t*-butyl, 5-trifluoromethyl)anilide	propyl	alkene
**7**	(2-*t*-butyl, 5-trifluoromethyl)anilide	H	alkyl
**8**	(2,5-di-trifluoromethyl)anilide	4-fluoropropane	alkyl
**9**	(2,5-di-trifluoromethyl)anilide	3-trifluoromethyl ethane	alkyl
**10**	(2,5-di-bromo)anilide	H	alkyl
**11**	adamantylamine	H	alkyl
**12**	(4-fluoro)diphenylmethyl[carbamoyl]	H	alkene
**13**	(4-fluoro)diphenylmethyl[carbamoyl]	propyl	alkene
**14**	(4-fluoro)diphenylmethyl[carbamoyl]	H	alkyl
**15**	(4-fluoro, 4-trifluoromethyl)-diphenylmethyl[carbamoyl]	H	alkene
**16**	(4-fluoro, 4-trifluoromethyl)-diphenylmethyl[carbamoyl]	propyl	alkene
**17**	(4-fluoro, 4-trifluoromethyl)-diphenylmethyl[carbamoyl]	H	alkyl
**18**	(4-fluorophenyl, pyridyl)-diphenylmethyl[carbamoyl]	H	alkene
**19**	(4-fluoro, 4-trifluoromethoxy)-diphenylmethyl[carbamoyl]	H	alkene
**20**	(4-fluoro, 4-trifluoromethoxy)-diphenylmethyl[carbamoyl]	H	alkyl
**21**	(4-fluoro, 4-trifluoromethoxy)-diphenylmethyl[carbamoyl]	H	alkyl
**(21)**	Additional Modification	3-hydroxy	
**22**	(4-difluoromethoxy)-diphenylmethyl[carbamoyl]	H	alkene
**23**	(4-difluoromethoxy)-diphenylmethyl[carbamoyl]	H	alkyl
**24**	(4-difluoromethoxy)-diphenylmethyl[carbamoyl]	propyl	alkyl

**25**	(4-trifluoromethyl)-diphenylmethyl[carbamoyl]	H	alkene
**26**	(4-trifluoromethyl)-diphenylmethyl[carbamoyl]	H	alkyl
**27**	(4-trifluoromethyl)-diphenylmethyl[carbamoyl]	propyl	alkyl
**28**	(4-trifluoromethoxy)-diphenylmethyl[carbamoyl]	H	alkene
**29**	(4-trifluoromethoxy)-diphenylmethyl[carbamoyl]	H	alkyl
**30**	(4-trifluoromethoxy)-diphenylmethyl[carbamoyl]	H	alkyl
**(30)**	Additional Modification	3-amino	
**31**	(4-trifluoromethoxy)-diphenylmethyl[carbamoyl]	acetyl	alkyl
**32**	(4-trifluoromethoxy)-diphenylmethyl[carbamoyl]	H	alkyl
**(32)**	Additional Modification	3-hydroxy	
**33**	(4-trifluoromethoxy)-diphenylmethyl[carbamoyl]	6-C (not aza)	alkene
**34**	(4-trifluoromethoxy)-diphenylmethyl[carbamoyl]	6-C (not aza)	alkyl
**35**	(4-trifluoromethoxy)-diphenylmethyl[carbamoyl]	propyl	alkyl
**36**	(4-trifluoromethoxy)-diphenylmethyl[carbamoyl]	propyl	alkene
**37**	(4-trifluoromethoxy)-diphenylmethyl[carbamoyl]	ethyl dimethylamine	alkyl
**38**	(4-trifluoromethoxy)-diphenylmethyl[carbamoyl]	methyl cyclopropyl	alkyl
**39**	(4-trifluoromethoxy)-diphenylmethyl[carbamoyl]	heptane	alkyl
**40**	(4-trifluoromethoxy)-diphenylmethyl[carbamoyl]	propyl	alkyl
**(40)**	Additional Modification	3-[2-(3-but-3-ynyldiazirin-3-yl) acetate]	
**41**	(4-trifluoromethoxy)-diphenylmethyl[carbamoyl]	4-fluoropropane	alkyl
**42**	(4-trifluoromethoxy)-diphenylmethyl[carbamoyl]	3-trifluoromethyl ethane	alkyl
**43**	4-carbamoyl-benzophenone	H	alkene
**44**	(4-carbamoyl(4’-(pent-4-ynamido)benzoyl)phenone)	H	alkene

Table S2. Overview of 6-azasteroids – activity & cytotoxicity

Cmpd	Structure	*Mtb* MIC Alone	Fold Δ of BDQ MIC		Mammalian Cell IC_{50} (μM)	
			Mtb	***Mm***	**HepG2**	**THP1**
**1**	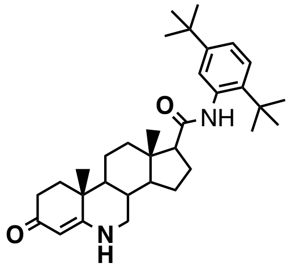	40	16	ND[a]	ND	17.7
**2**	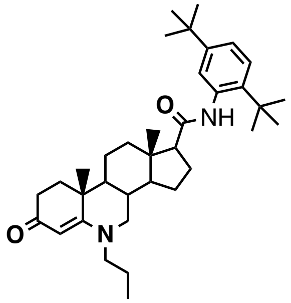	37	8	9	21	24
**3**	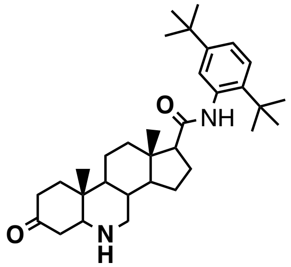	10	ND	ND	16.94	14.2
**4**	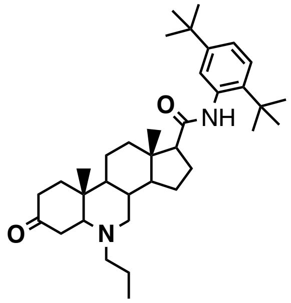	20	ND	ND	ND	73
**5**	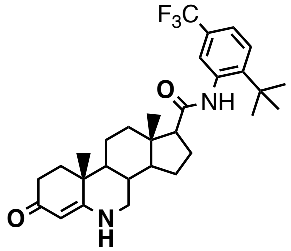	>20	2	ND	60	ND

**6**	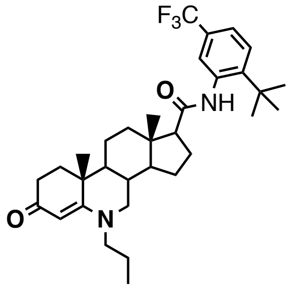	ND (*Mm* >20)	ND	3	71	ND
**7**	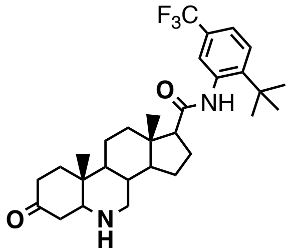	30	ND	ND	12.1	ND
**8**	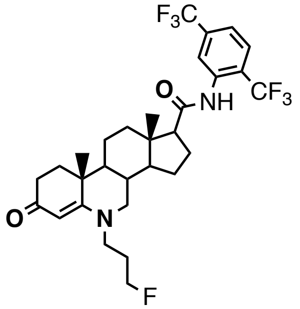	ND (*Mm* >20)	ND	7	>100	ND
**9**	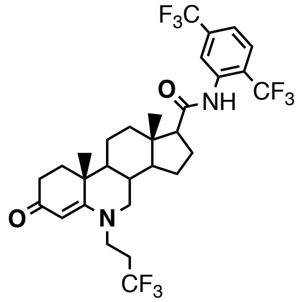	ND (*Mm* >20)	ND	4	>100	ND
**10**	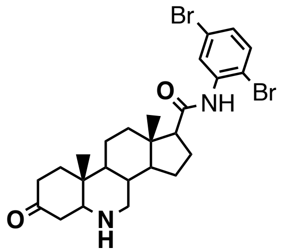	30	ND	ND	7.6	ND
**11**	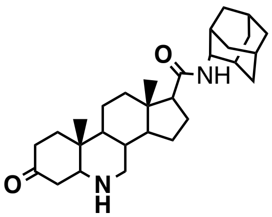	>80	ND	6	89.3	ND

**12**	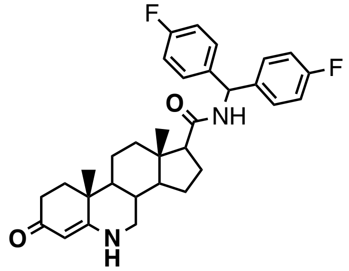	31	32	7	31.4	24.2
**13**	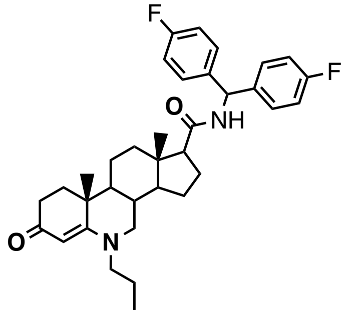	>20	128	ND	>100	ND
**14**	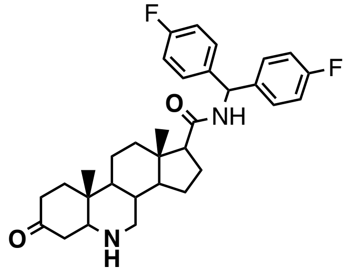	80	ND	ND	14.22	ND
**15**	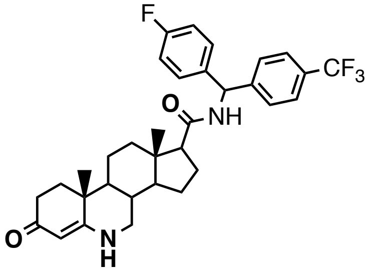	>20	64	ND	26	ND
**16**	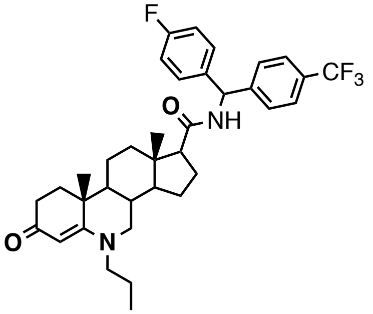	>20	64	ND	ND	ND

**17**	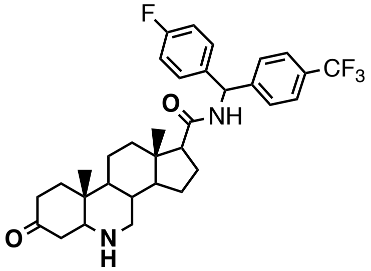	20	ND	ND	10.1	15.6
**18**	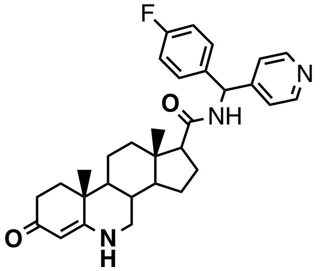	15	4	ND	>100	ND
**19**	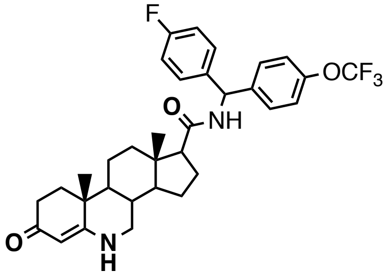	>20	32-64	ND	58.4	ND
**20**	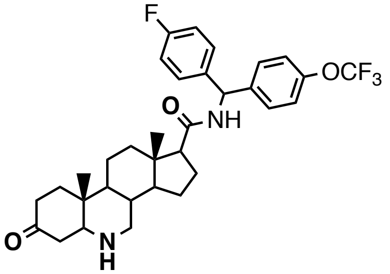	>20	>32	ND	5.7	ND
**21**	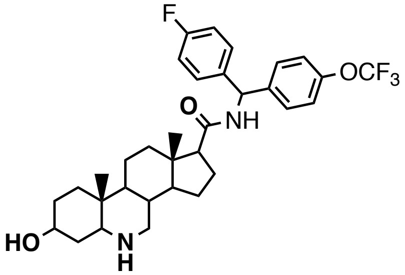	>20	>32	ND	ND	ND
**22**	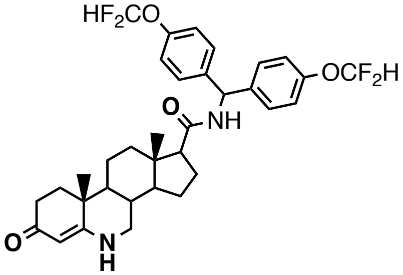	>20	>32	ND	ND	25.7

**23**	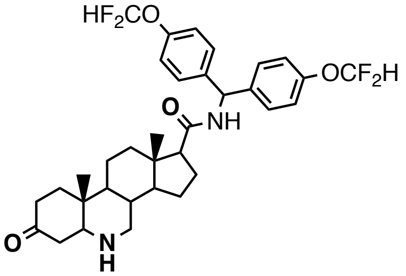	15	ND	ND	ND	3.3
**24**	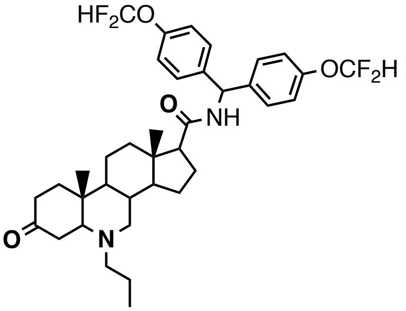	60	ND	25.5	ND	>100
**25**	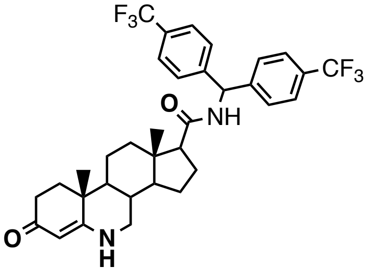	20	8	ND	75.69	ND
**26**	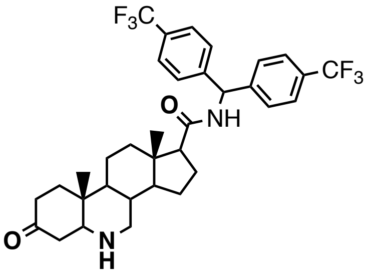	7.5	ND	ND	6	ND
**27**	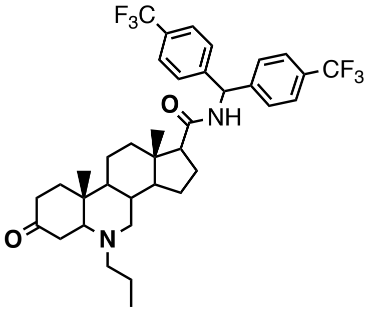	20	2	ND	ND	>100

**28**	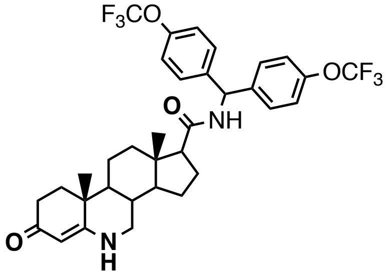	>20	>32	ND	ND	12.8
**29**	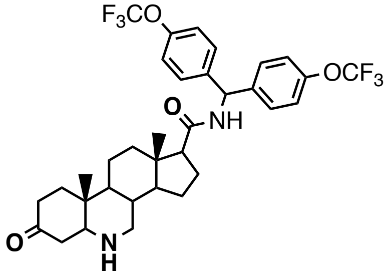	5	ND	ND	ND	4.5
**30**	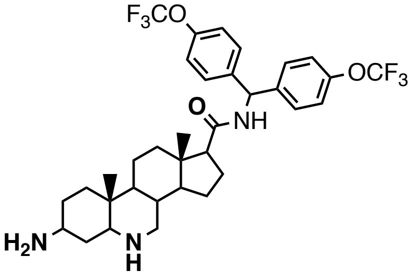	80	ND	ND	2.7	ND
**31**	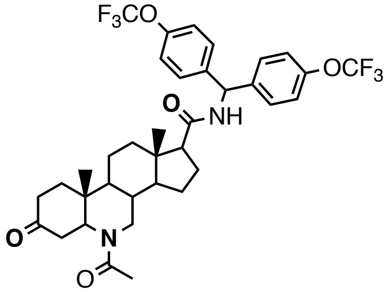	>80	1	3.5	>100	ND
**32**	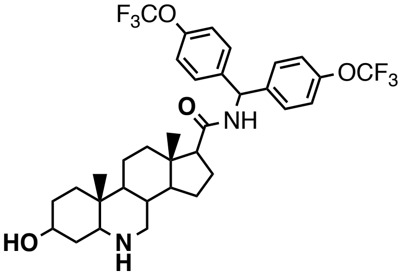	5	ND	ND	5.75	ND
**33**	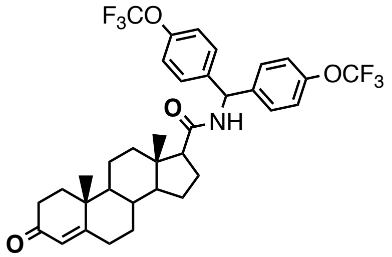	>80	1	ND	>100	ND

**34**	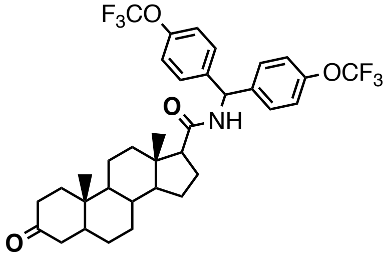	>80	1	ND	>100	ND
**35**	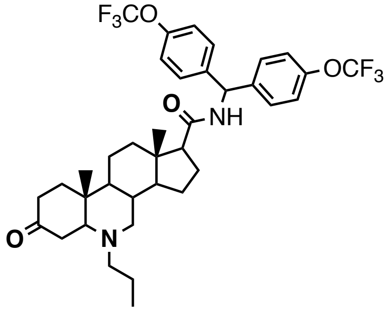	>80	8	8	ND	>100
**36**	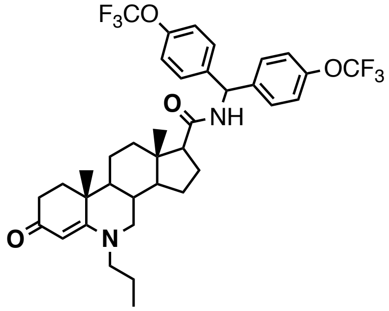	>40	ND	1.6	ND	>100
**37**	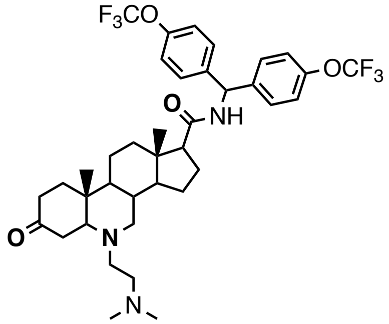	5	ND	ND	ND	4.1
**38**	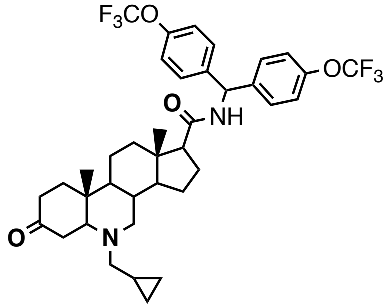	80	ND	ND	ND	10

**39**	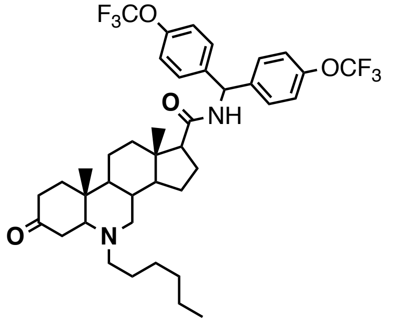	80	ND	1.5	ND	>100
**40**	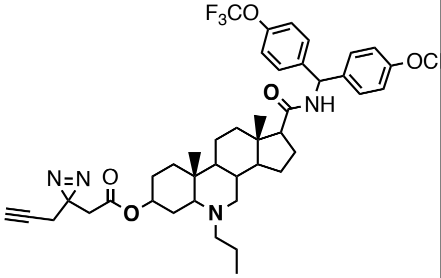	ND (*Mm* >20)	ND	3.7	>100	ND
**41**	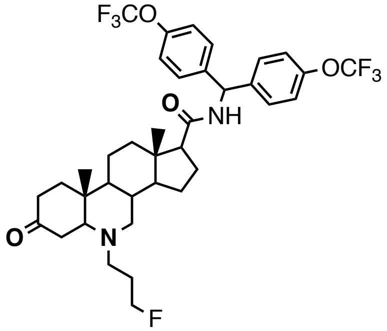	ND (*Mm* >20)	ND	5	>100	ND
**42**	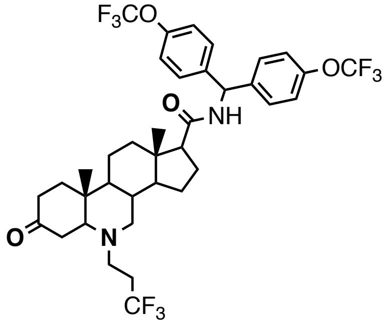	ND (*Mm* >20)	ND	1	>100	ND
**43**	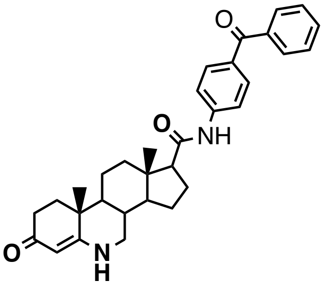	ND (*Mm* >20)	8	2	>100	ND

**44**	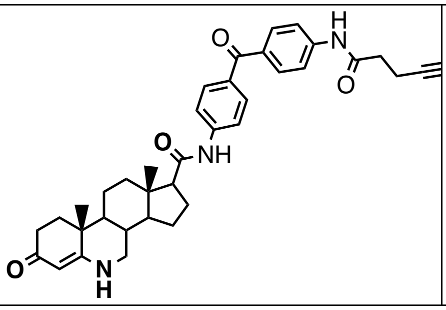	ND (*Mm* >20)	ND	1.1-2.5	ND	ND

[a]ND: not determined.

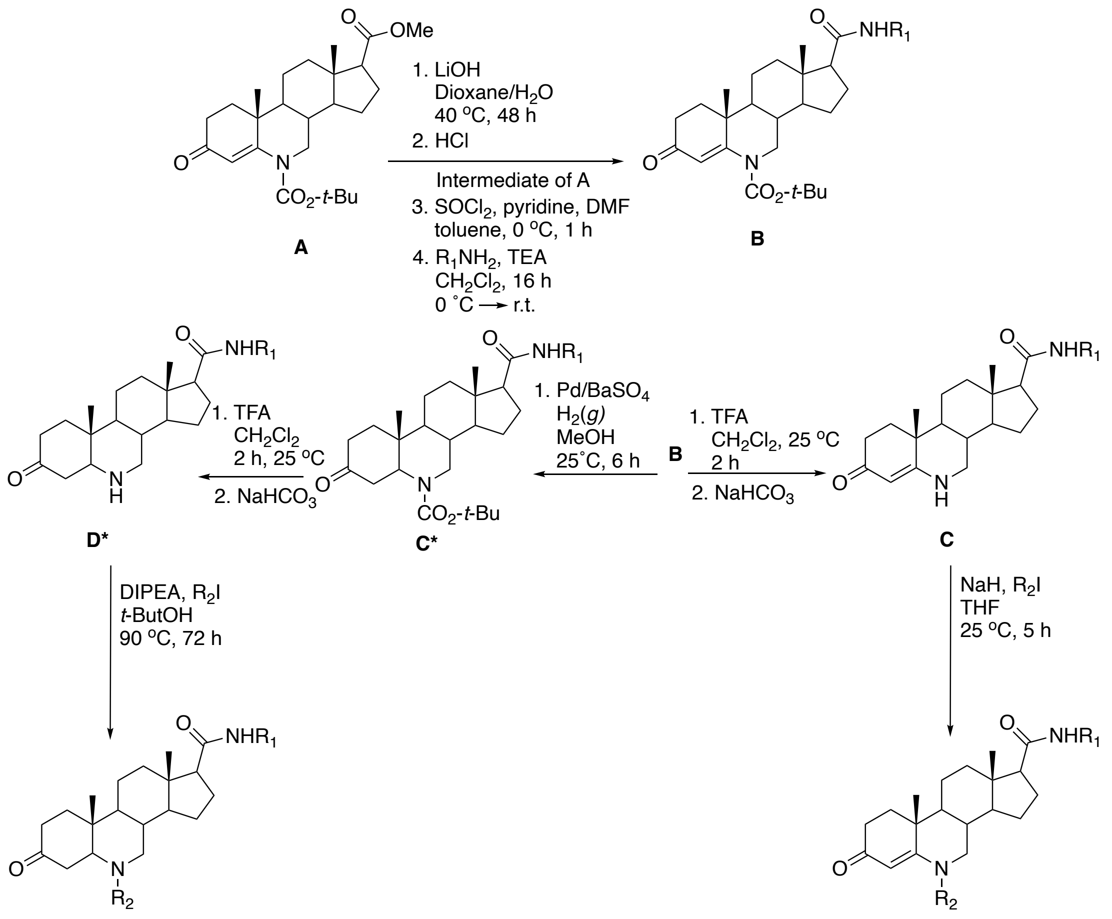

Figure S1. Synthesis of 6-azasteroids.

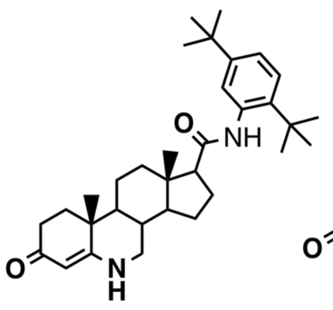

1

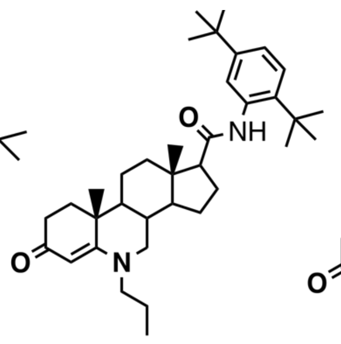

2

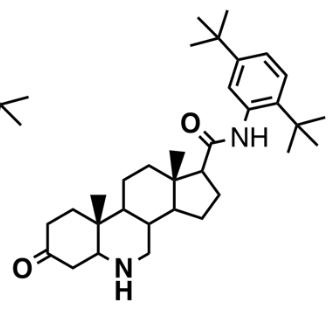

3

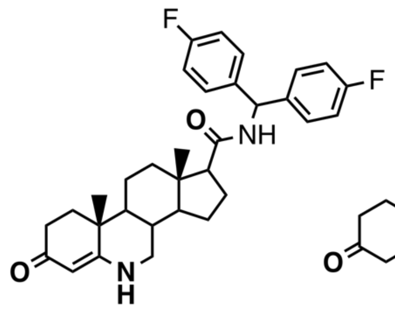

12

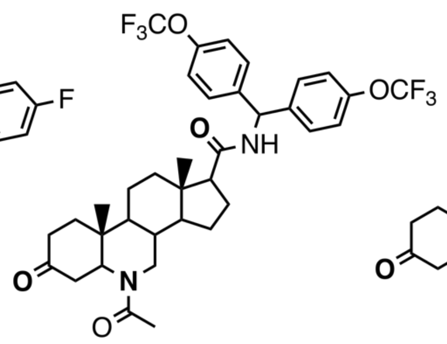

31

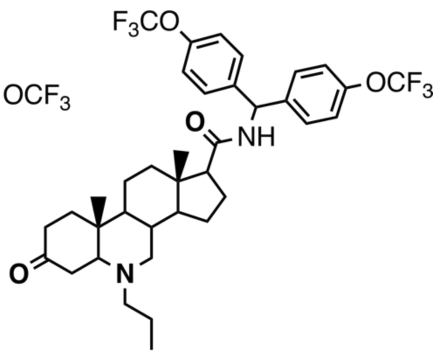

35

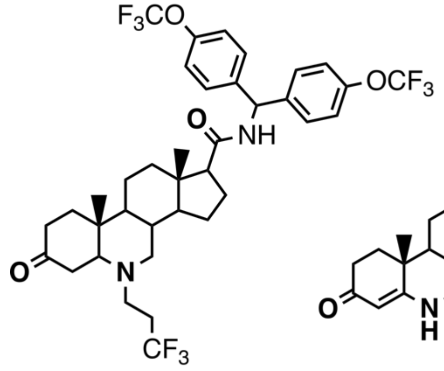

42

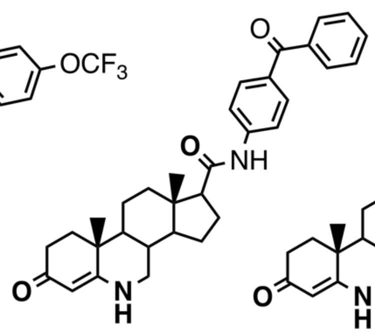

43

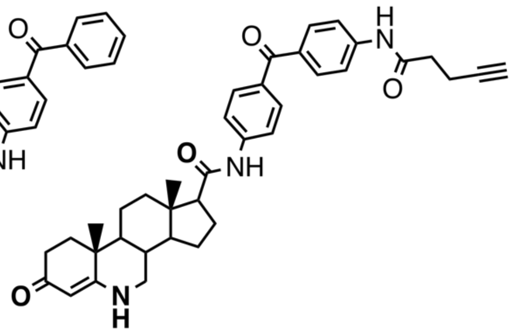

44

Figure S2. Structures of 6-azasteroids tested for inhibition of *Mtb* 3β-hydroxysteroid dehydrogenase.

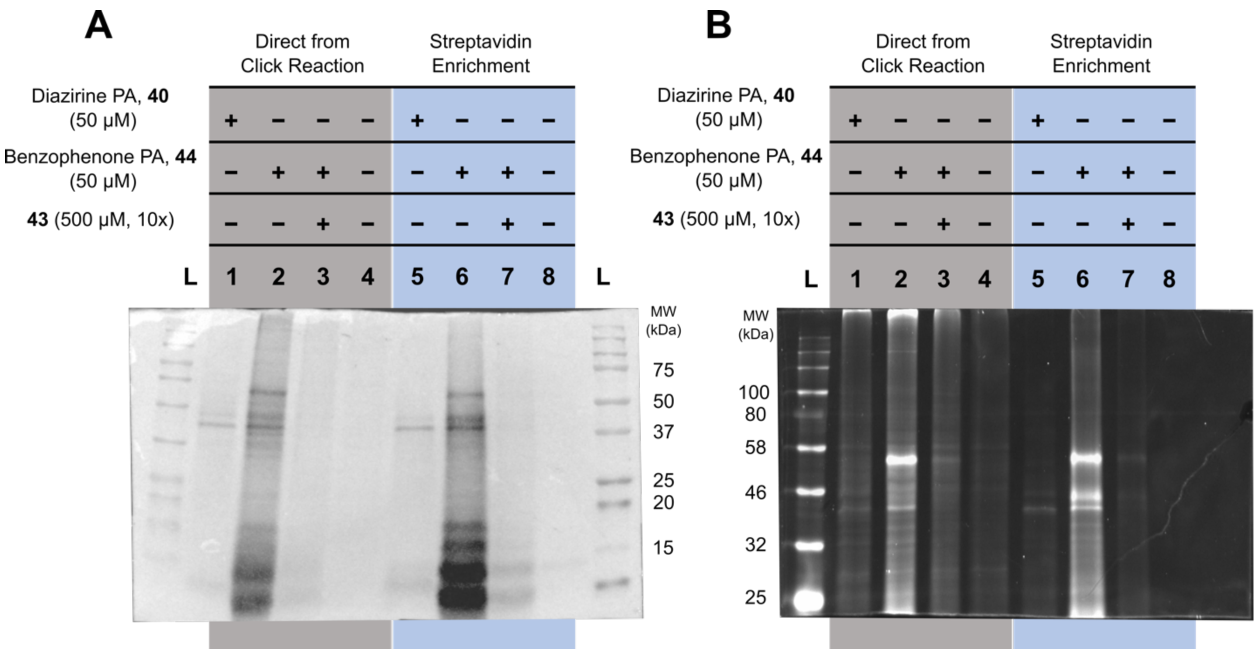

Figure S3. (A) Full western blot & (B) full fluorescence image of whole cell lysate interaction assay, proteins enriched via azido-PEG3-TAMRA-biotin. Probe **40 (**Lane 1 & 5), **44 (**Lane 2 & 6), **44** with an excess of **43** (Lane 3 & 7), or DMSO (Lane 4 & 8) after click conjugation with azido-PEG3-TAMRA-biotin and streptavidin agarose bead-mediated enrichment.

A

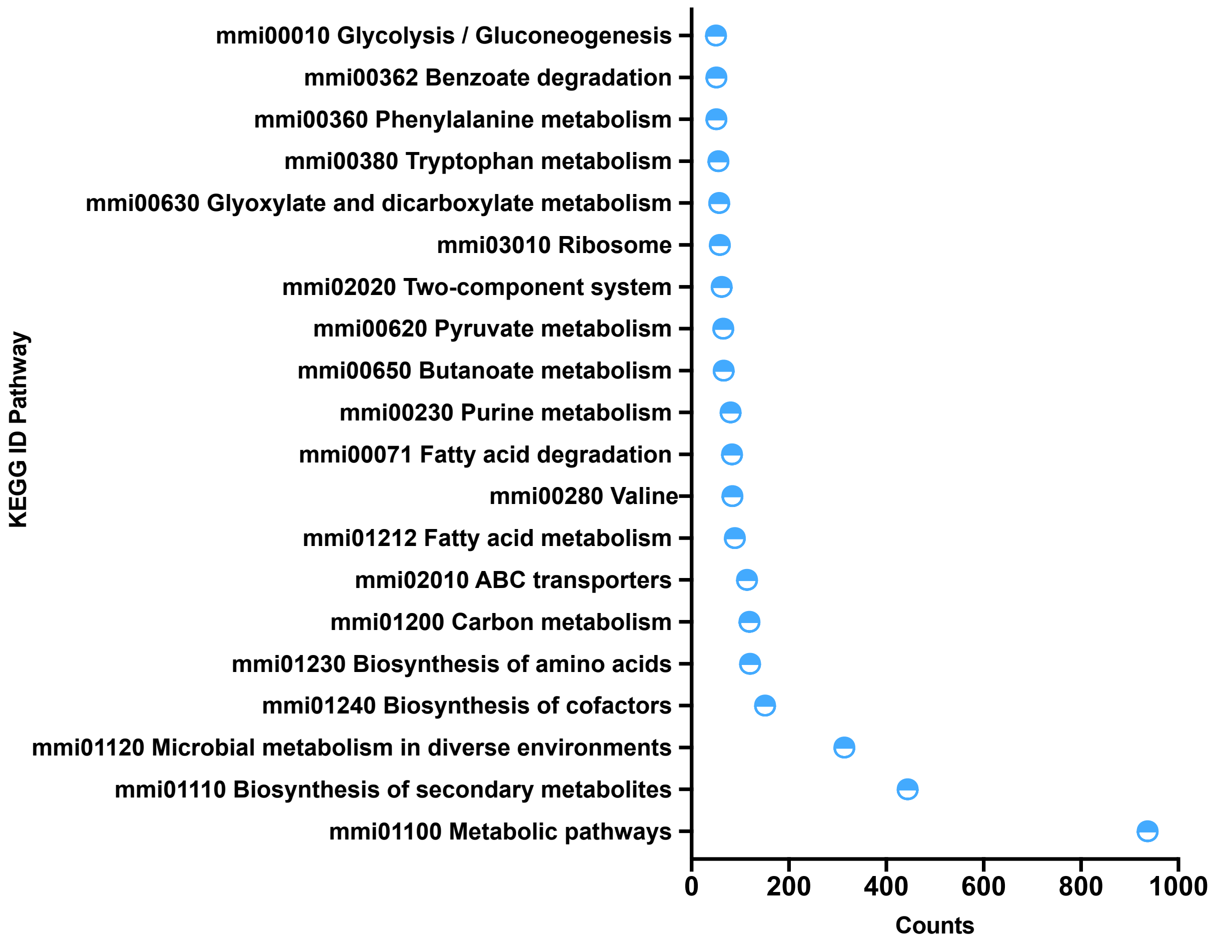
KEGG ID Pathway
mmi00010 Glycolysis / Gluconeogenesis
mmi00362 Benzoate degradation
mmi00360 Phenylalanine metabolism
mmi00380 Tryptophan metabolism
mmi00630 Glyoxylate and dicarboxylate metabolism
mmi03010 Ribosome
mmi02020 Two-component system
mmi00620 Pyruvate metabolism
mmi00650 Butanoate metabolism
mmi00230 Purine metabolism
mmi00071 Fatty acid degradation
mmi00280 Valine
mmi01212 Fatty acid metabolism
mmi02010 ABC transporters
mmi01200 Carbon metabolism
mmi01230 Biosynthesis of amino acids
mmi01240 Biosynthesis of cofactors
mmi01120 Microbial metabolism in diverse environments
mmi01110 Biosynthesis of secondary metabolites
mmi01100 Metabolic pathways
0
200
400
600
800
1000
Counts

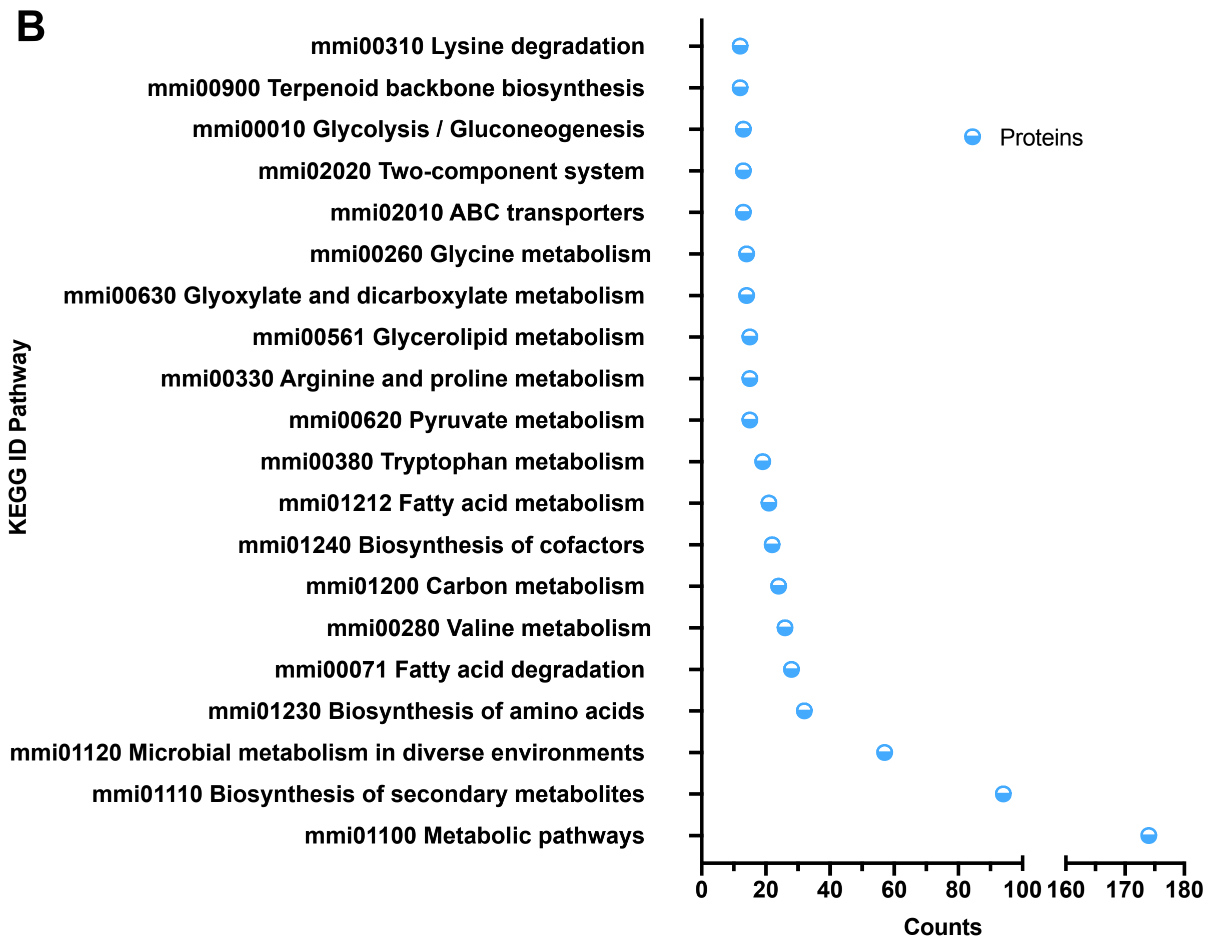
B
mmi00310 Lysine degradation
mmi00900 Terpenoid backbone biosynthesis
mmi00010 Glycolysis / Gluconeogenesis
mmi02020 Two-component system
mmi02010 ABC transporters
mmi00260 Glycine metabolism
mmi00630 Glyoxylate and dicarboxylate metabolism
mmi00561 Glycerolipid metabolism
mmi00330 Arginine and proline metabolism
mmi00620 Pyruvate metabolism
mmi00380 Tryptophan metabolism
mmi01212 Fatty acid metabolism
mmi01240 Biosynthesis of cofactors
mmi01200 Carbon metabolism
mmi00280 Valine metabolism
mmi00071 Fatty acid degradation
mmi01230 Biosynthesis of amino acids
mmi01120 Microbial metabolism in diverse environments
mmi01110 Biosynthesis of secondary metabolites
mmi01100 Metabolic pathways
KEGG ID Pathway
Proteins
0
20
40
60
80
100
160
170
180
Counts

C

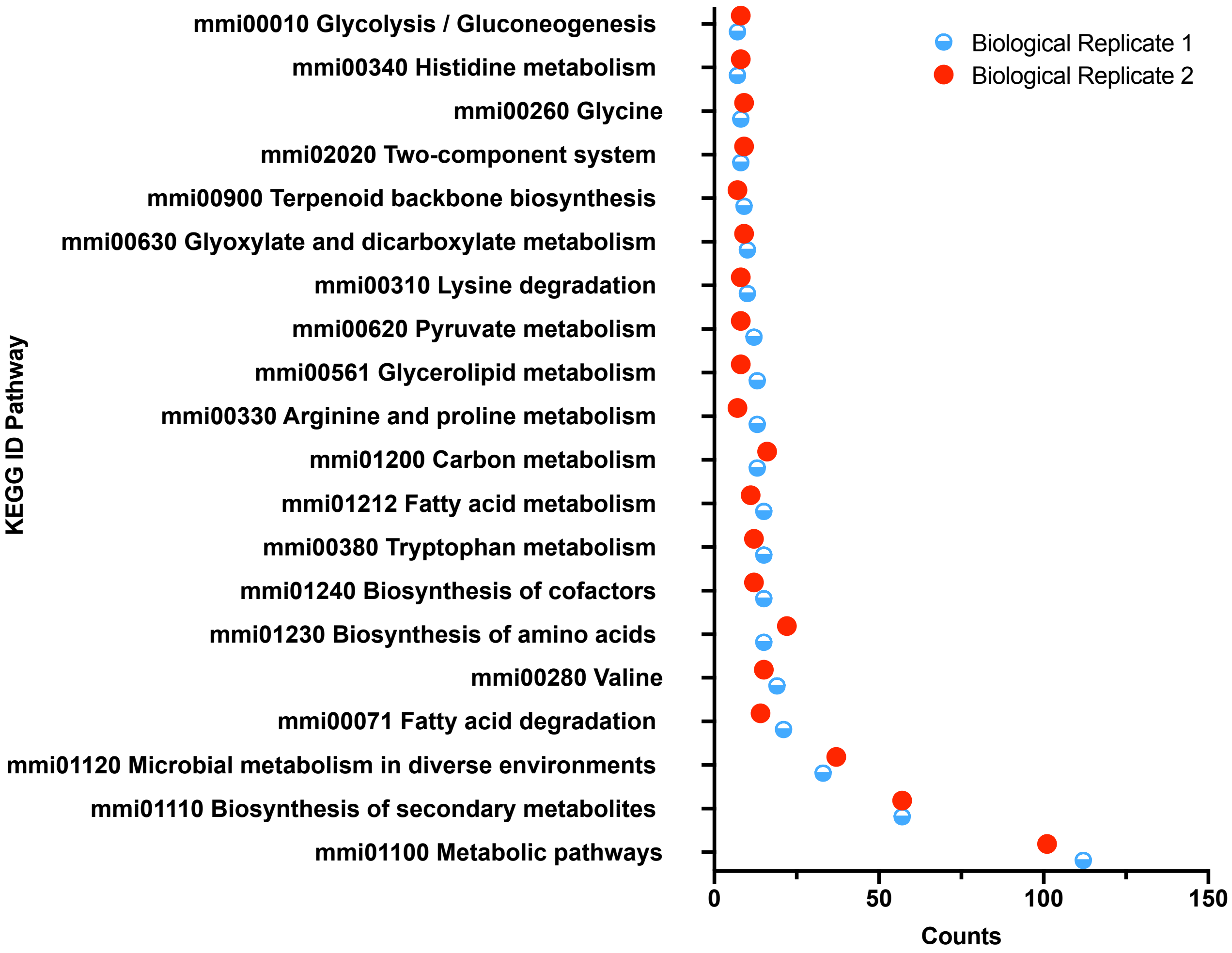

Figure S4. KEGG ID functional analysis of the entire *Mm* proteome (A) and the entire proteomics data set (B) by top 20 functional categories. (C) KEGG ID Functional Analysis of *Mm* Azasteroid Probe Enriched Interactome Proteins (top 20 functional categories).

BenzophenonePA

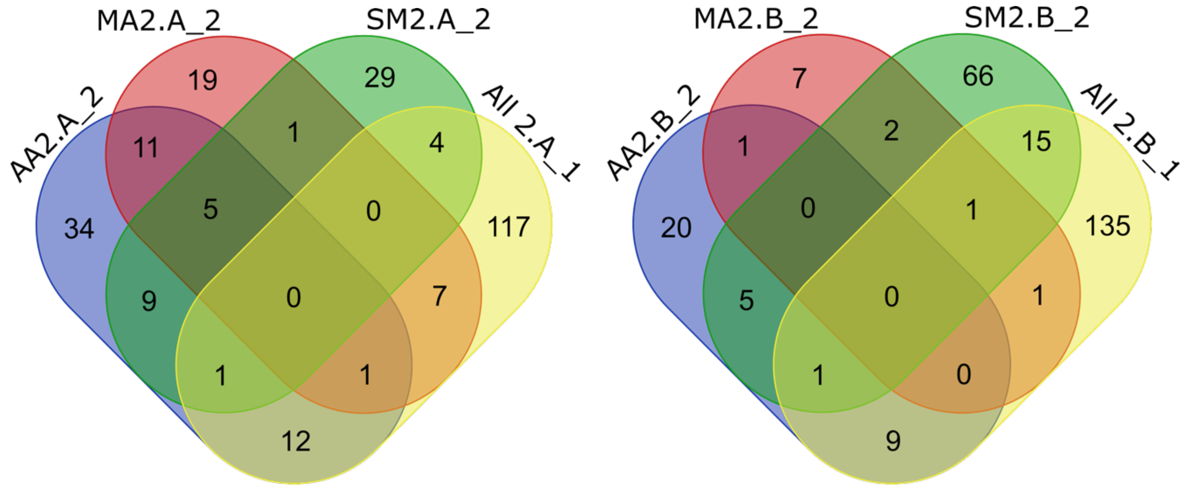

DiazirinePA

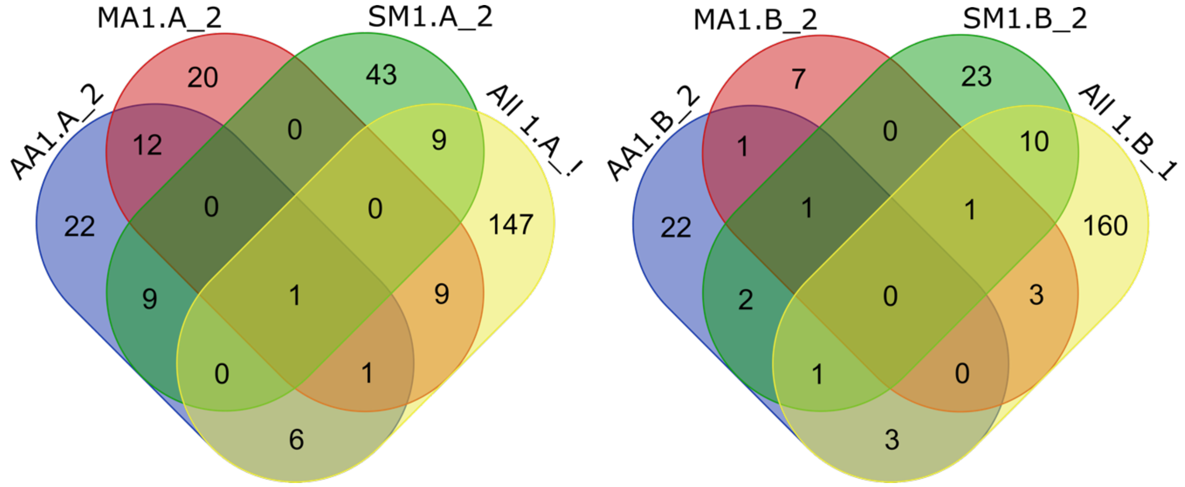

Figure S5. Enrichment methods comparison by Venn diagram
Enrichment Methods: AA: Azide Agarose Bead, MA: Azido-functionalized Magnetic Nanoparticles, SM: Streptavidin-functionalized Magnetic Nanoparticles. First, number: Probe, 1: Diazirine PA, 2: Benzophenone PA. Second, letter: 1:DMSO Control, 2:Linker Control. Third, number: 1: First Biological Replicate, 2: Second Biological Replicate.

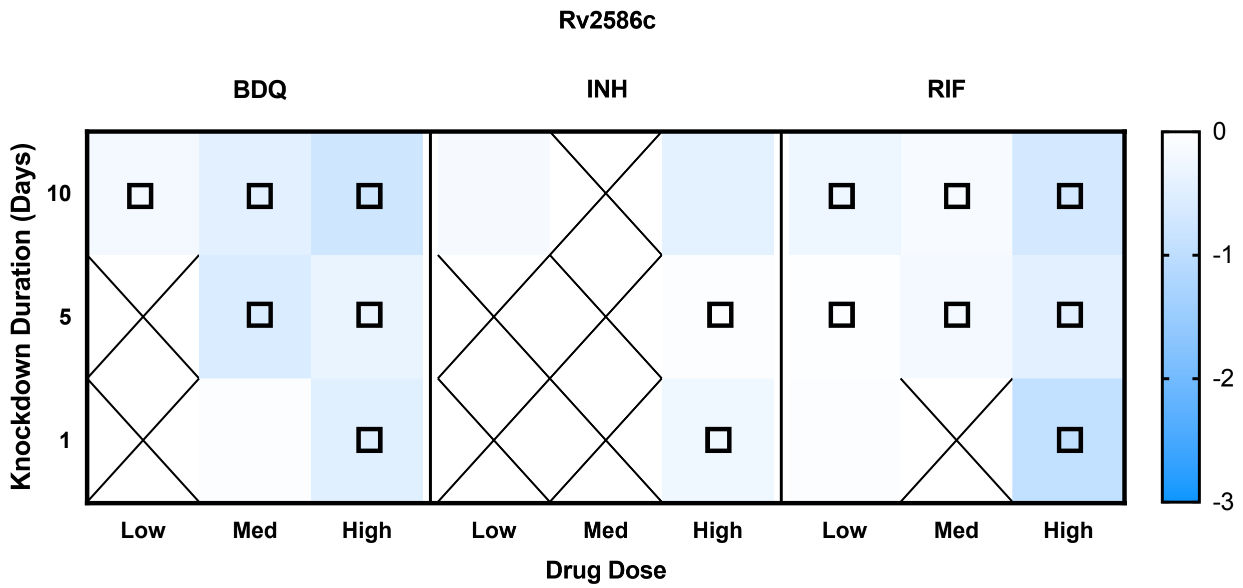
Rv2586c
BDQ
INH
RIF
Knockdown Duration (Days)
10
5
1
Low
Med
High
Low
Med
High
Low
Med
High
Drug Dose
0
-1
-2
-3

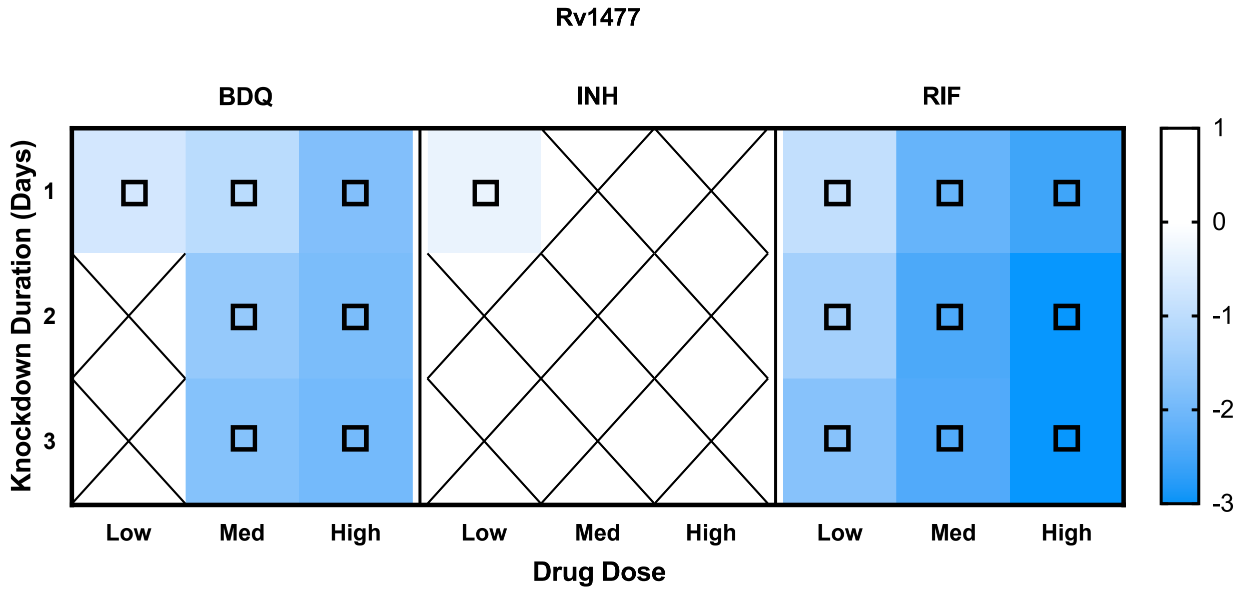
Rv1477
BDQ
INH
RIF
Knockdown Duration (Days)
1
2
3
Low
Med
High
Low
Med
High
Low
Med
High
Drug Dose
1
0
-1
-2
-3

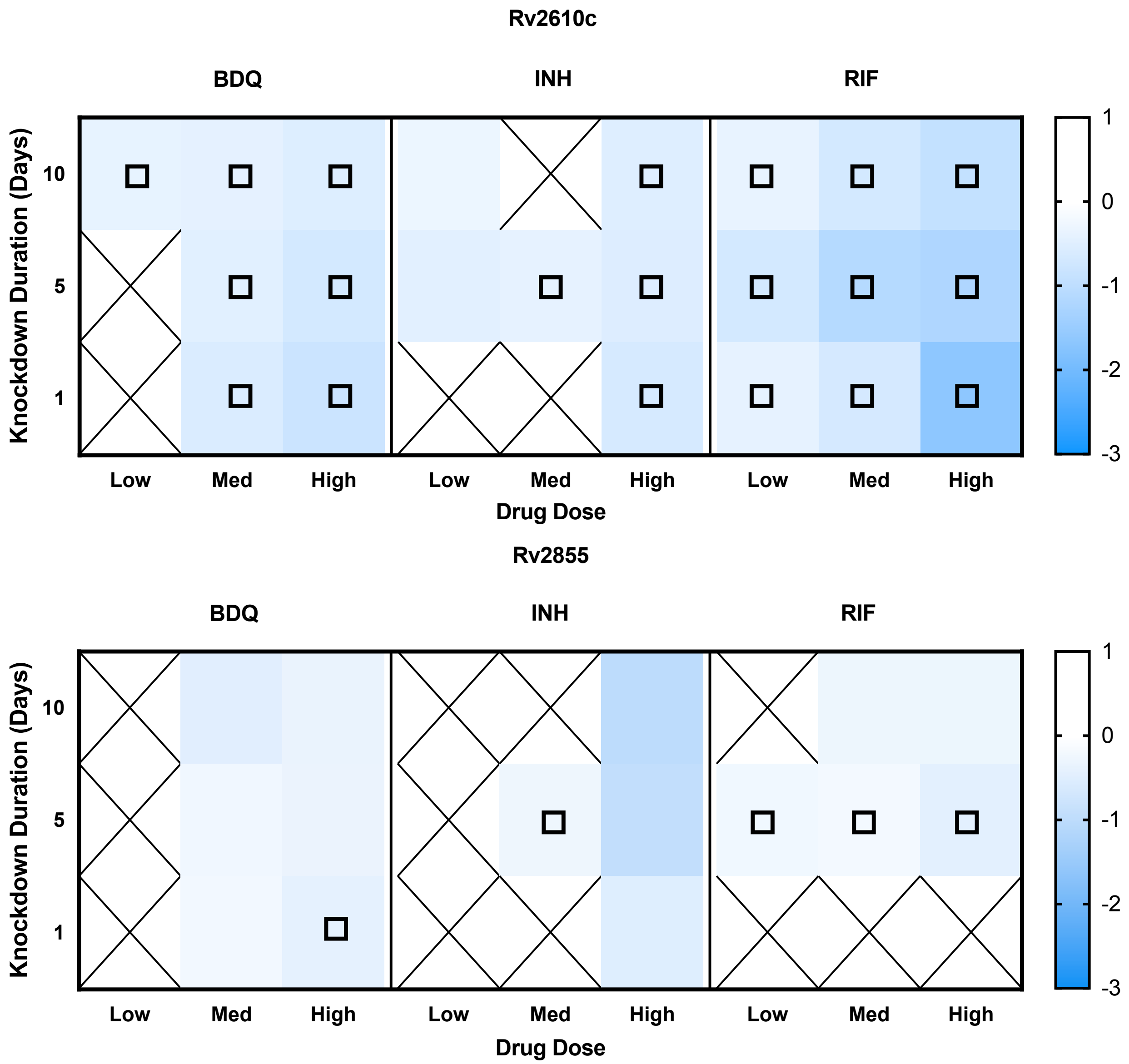
Rv2610c
BDQ
INH
RIF
Knockdown Duration (Days)
10
5
1
Low
Med
High
Low
Med
High
Low
Med
High
Drug Dose
1
0
-1
-2
-3
Rv2855
BDQ
INH
RIF
Knockdown Duration (Days)
10
5
1
Low
Med
High
Low
Med
High
Low
Med
High
Drug Dose
1
0
-1
-2
-3

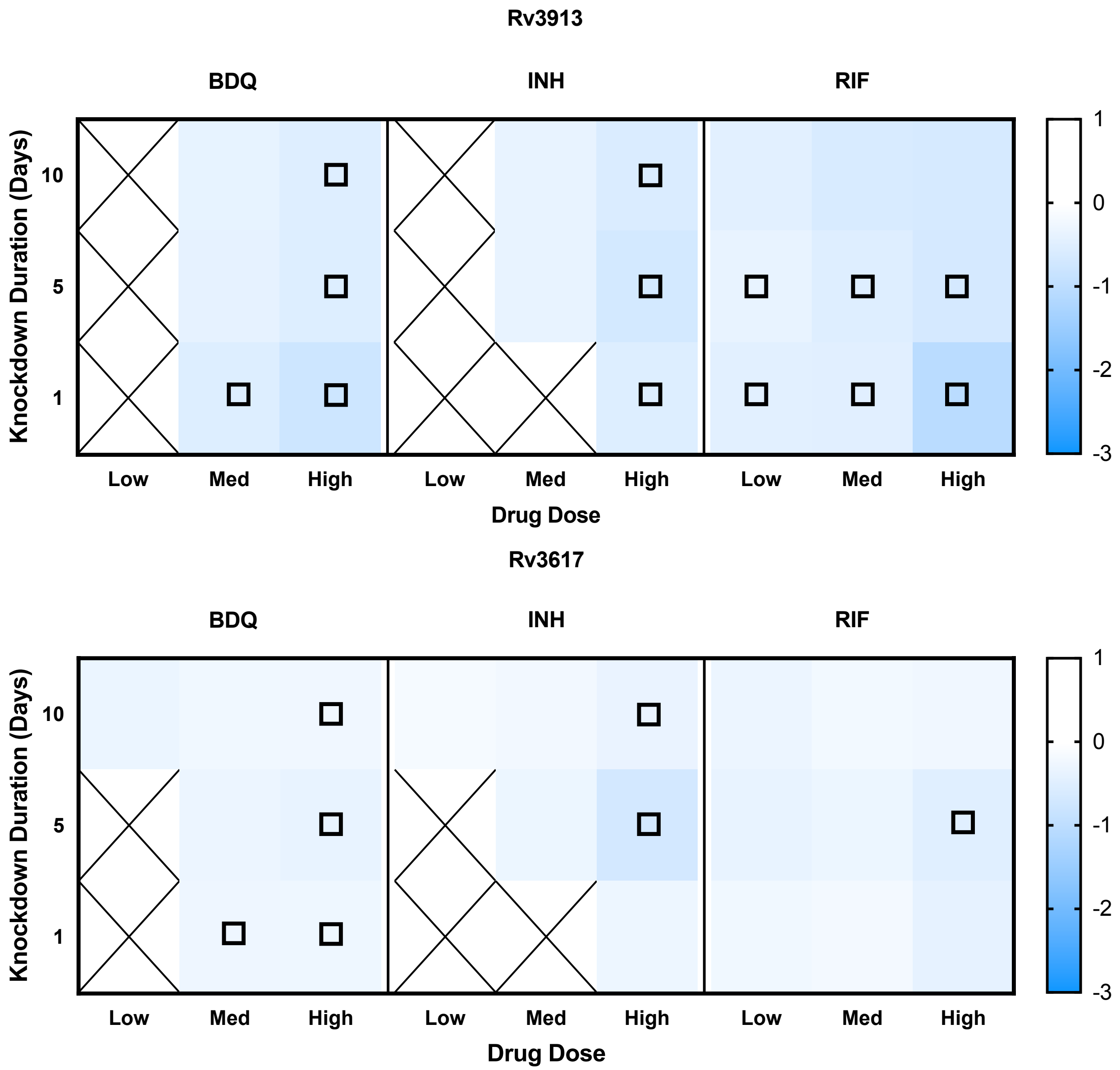

Rv3913
BDQ
INH
RIF
Knockdown Duration (Days)
10
5
1
Low
Med
High
Low
Med
High
Low
Med
High
Drug Dose
1
0
-1
-2
-3
Rv3617
BDQ
INH
RIF
Knockdown Duration (Days)
10
5
1
Low
Med
High
Low
Med
High
Low
Med
High
Drug Dose
1
0
-1
-2
-3

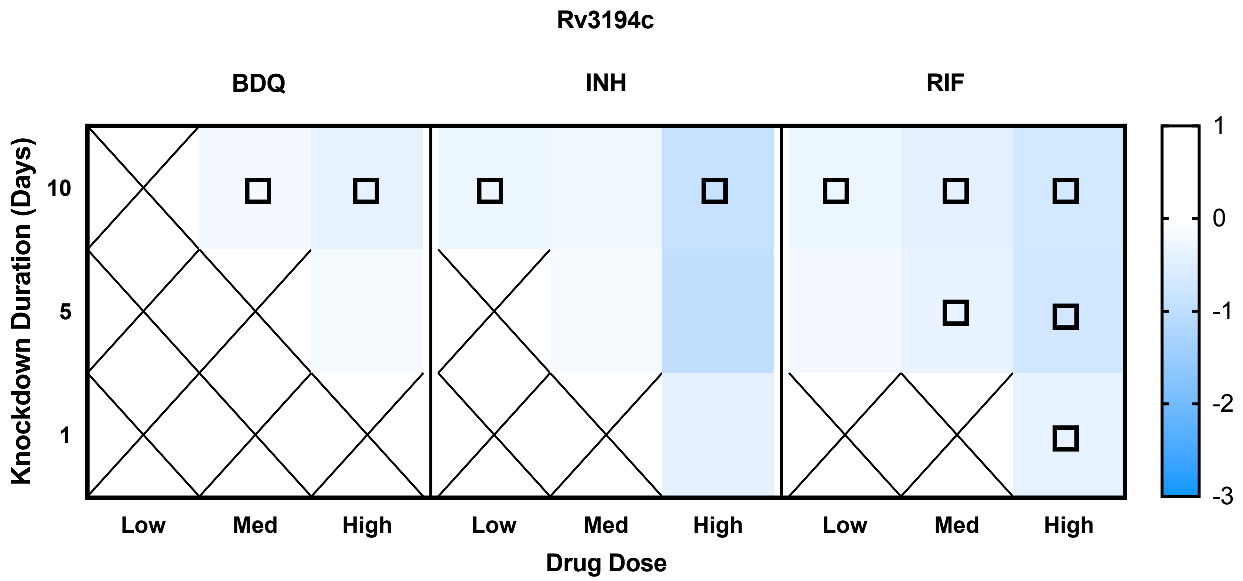

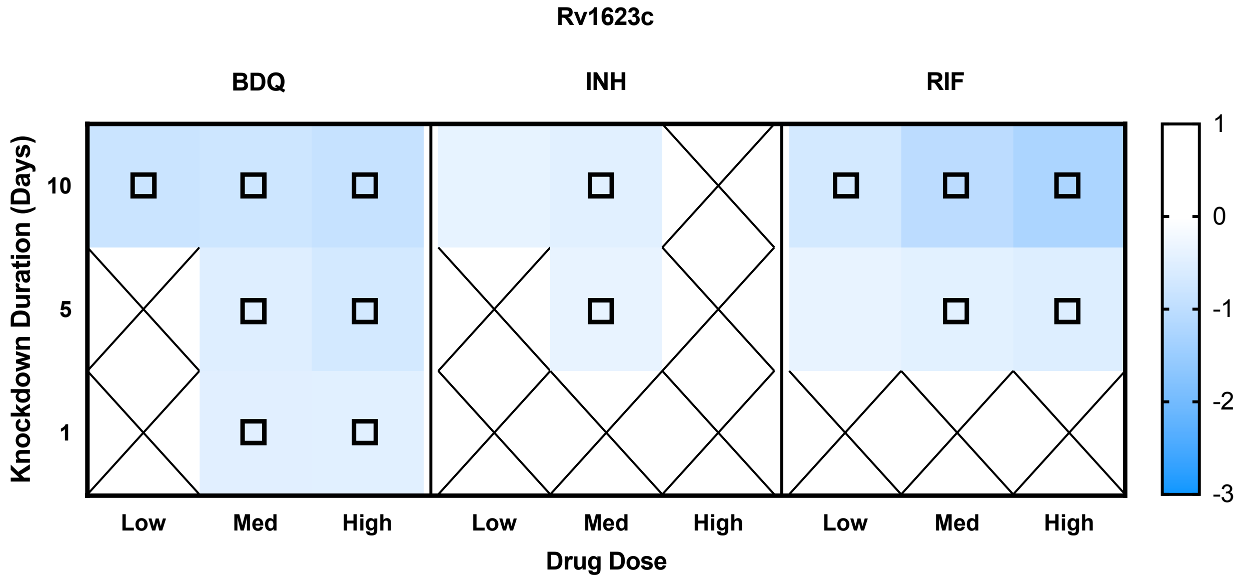

Figure S6. CRISPRi negative screen of selected genes against BDQ, INH, and RIF treatment which were hits of probes. Data was mined from Li *et al.*[1] and visualized heat map plots prepared in Graphpad Prism. Conditions which had a False Discovery Rate of 1 are denoted with an X. Conditions which had an FDR < 0.01 are demarcated with a square in the center of its position.

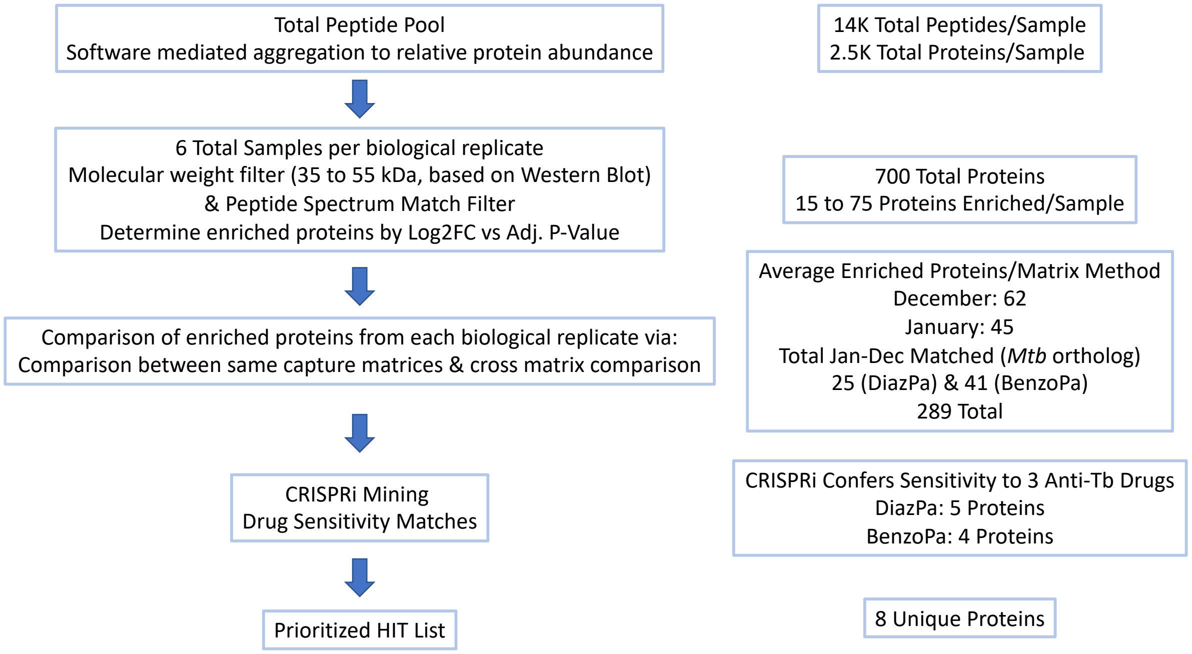

Figure S7. Overview of pull-down assay data analysis. The filtering methodology utilized to obtain the list of prioritized hits as targets of 6-azasteroid probes.

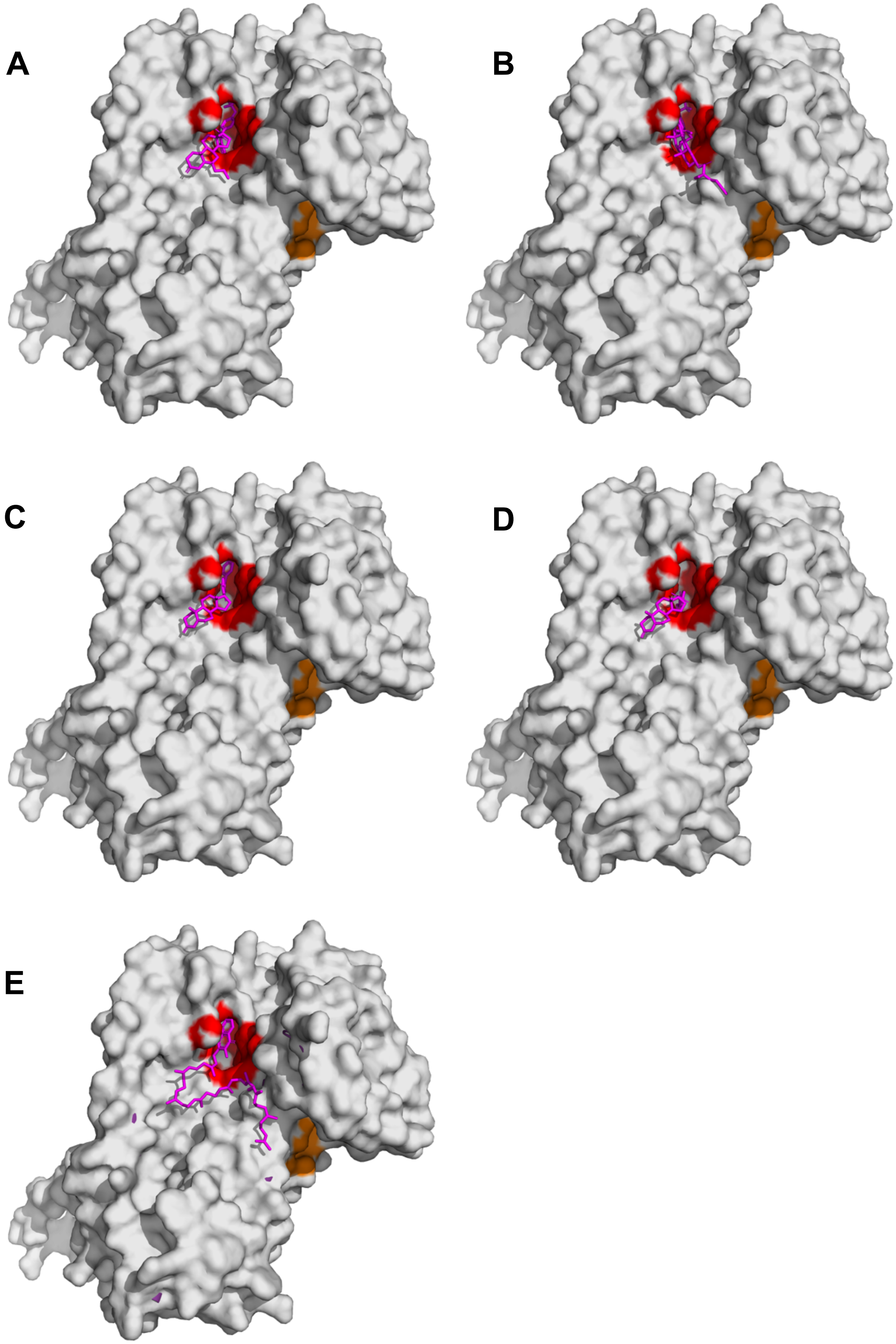

Figure S8. Docking of azasteroids to CydA (Rv1623c). (A) Compound **35.** (B) DiazirinePA **40**. (C) Compound **43** (D) BenzophenonePA **44**. (E) Menaquinone-9 (MQ9).

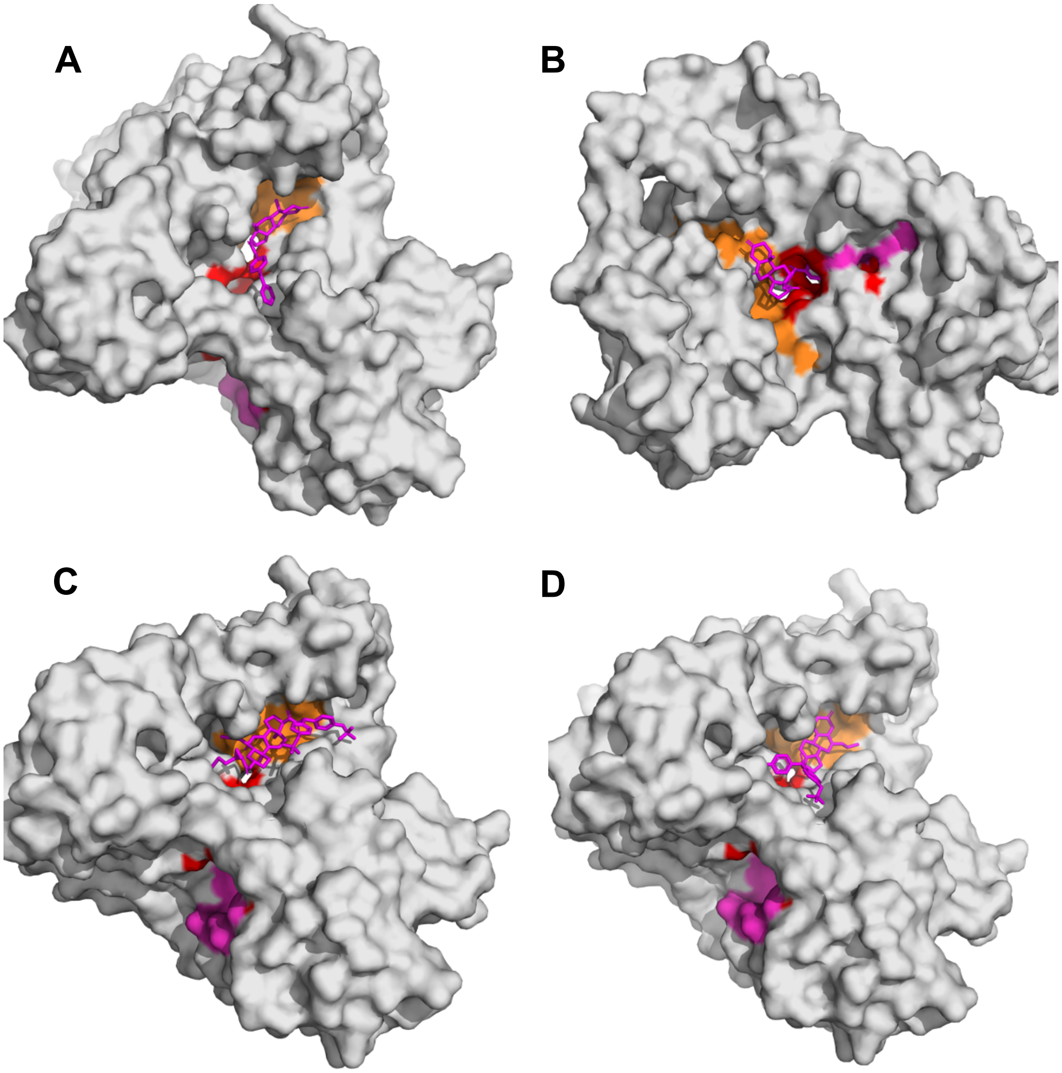

Figure S9. Docking of azasteroids to Mtr (Rv2855). (A) Compound **35.** (B) DiazirinePA **40**. (C) Compound **43** (D) BenzophenonePA **44**.

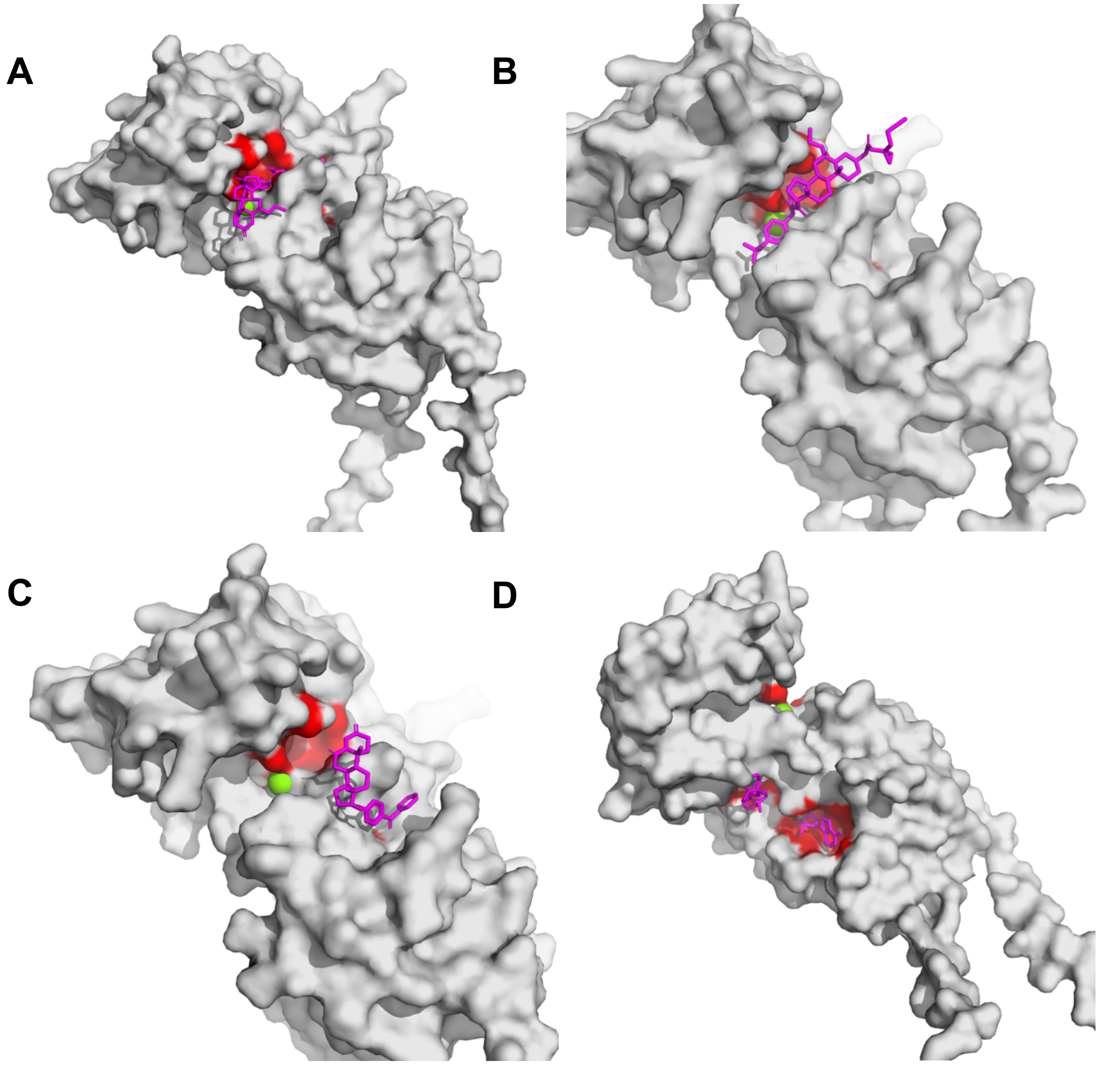

Figure S10. Docking of azasteroids to TrxB2 (Rv3913). (A) Compound **35.** (B) DiazirinePA **40**. (C) Compound **43** (D) BenzophenonePA **44**.

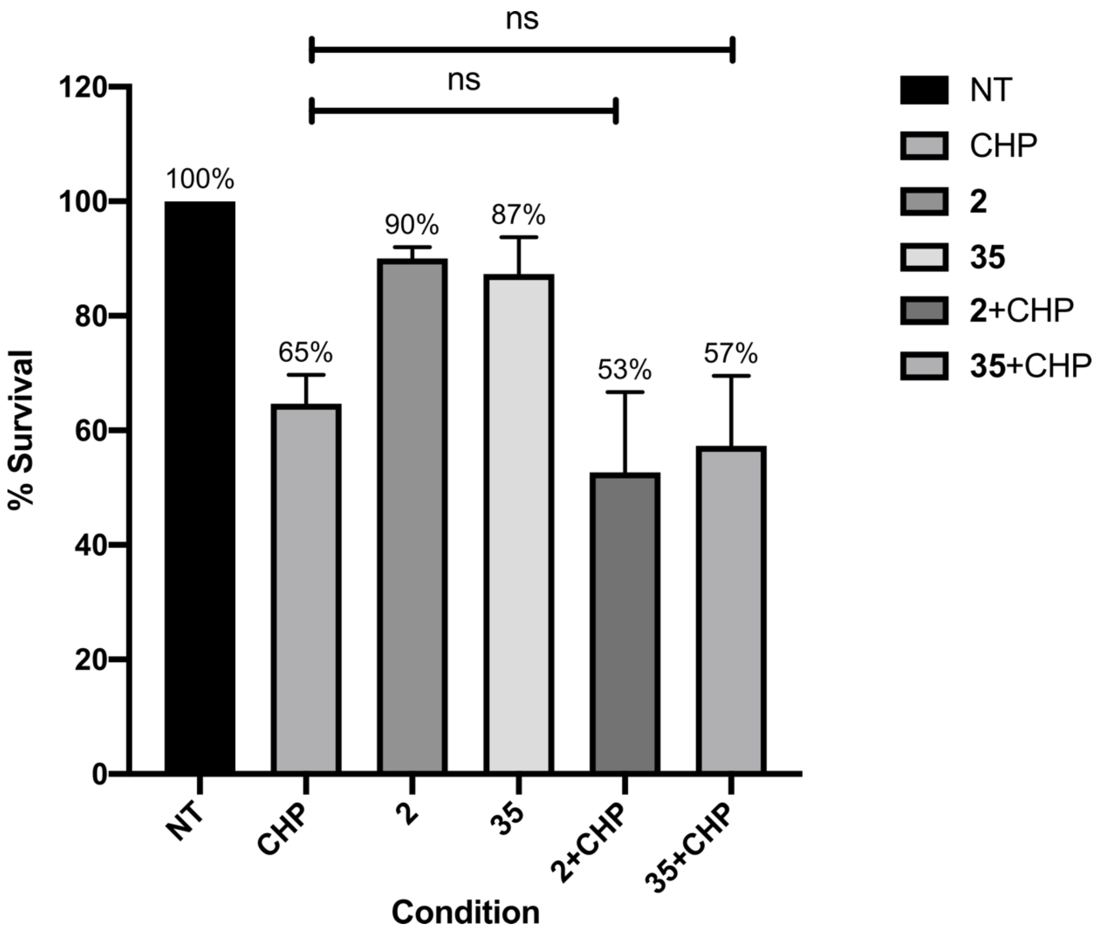

Figure S11. Survival of *Mtb* cultures treated with peroxide alone and in combination with 6-azasteroid. *Mtb* cultures were pre-incubated with 6-azasteroid or DMSO for 6 hours. Cultures were exposed to cumene hydroperoxide stress for 30 minutes and after workup were plated on 7H11 agarose plates for CFU counting to determine impacts on survival. Data are the mean of three technical replicates ± standard deviation.

A. Synthesis of Diazirine PA

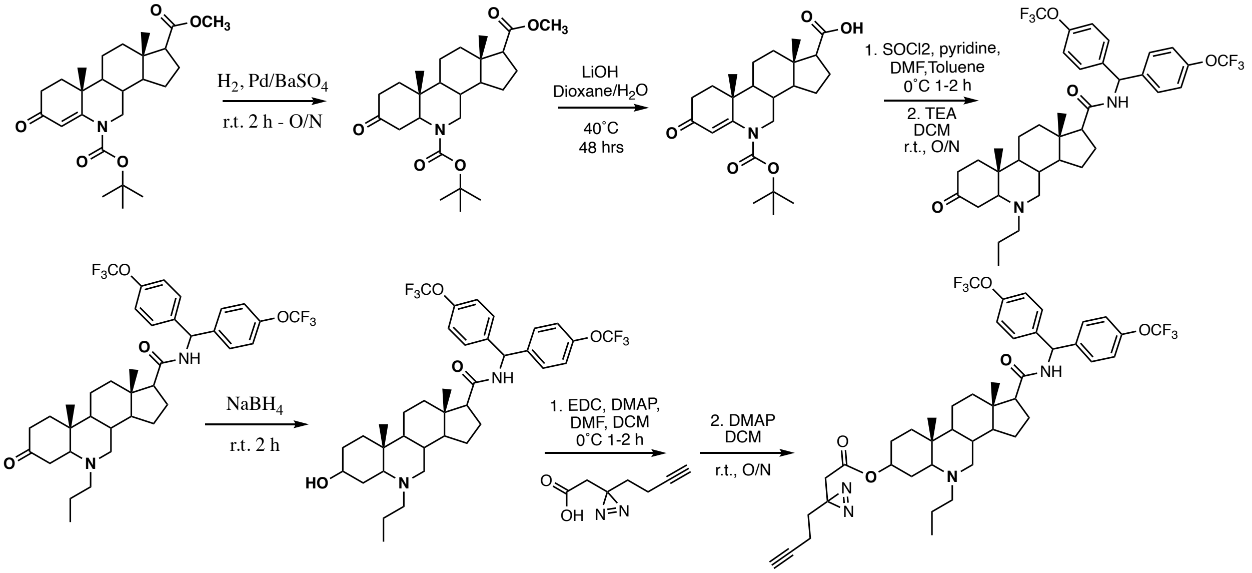

B. Synthesis of Benzophenone PA

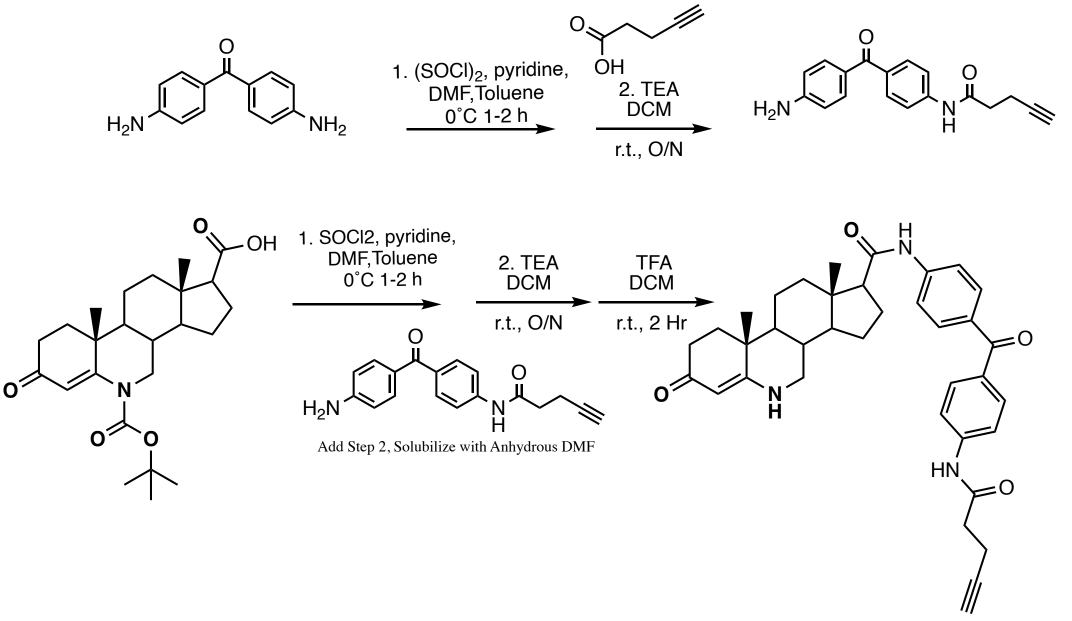

Figure S12. Synthesis of 6-azasteroid probes.

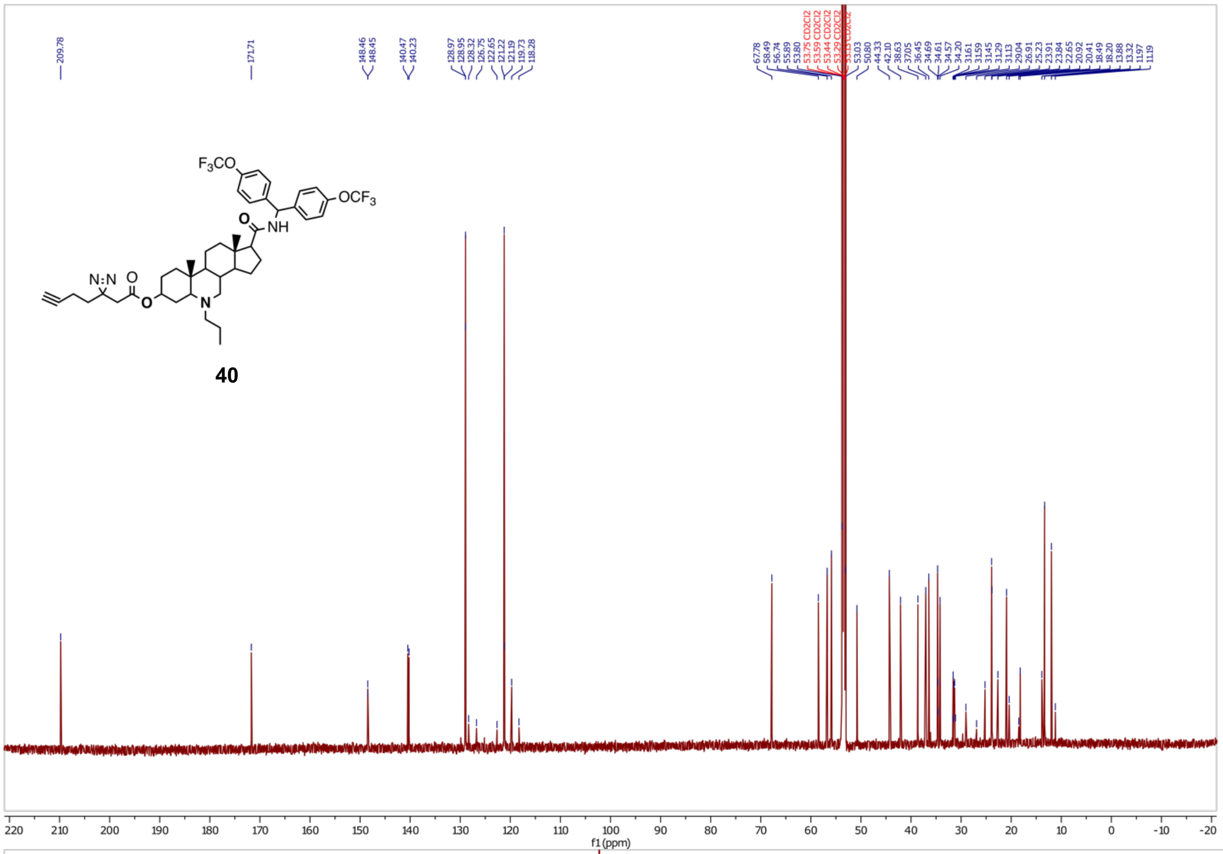
40
f1 (ppm)

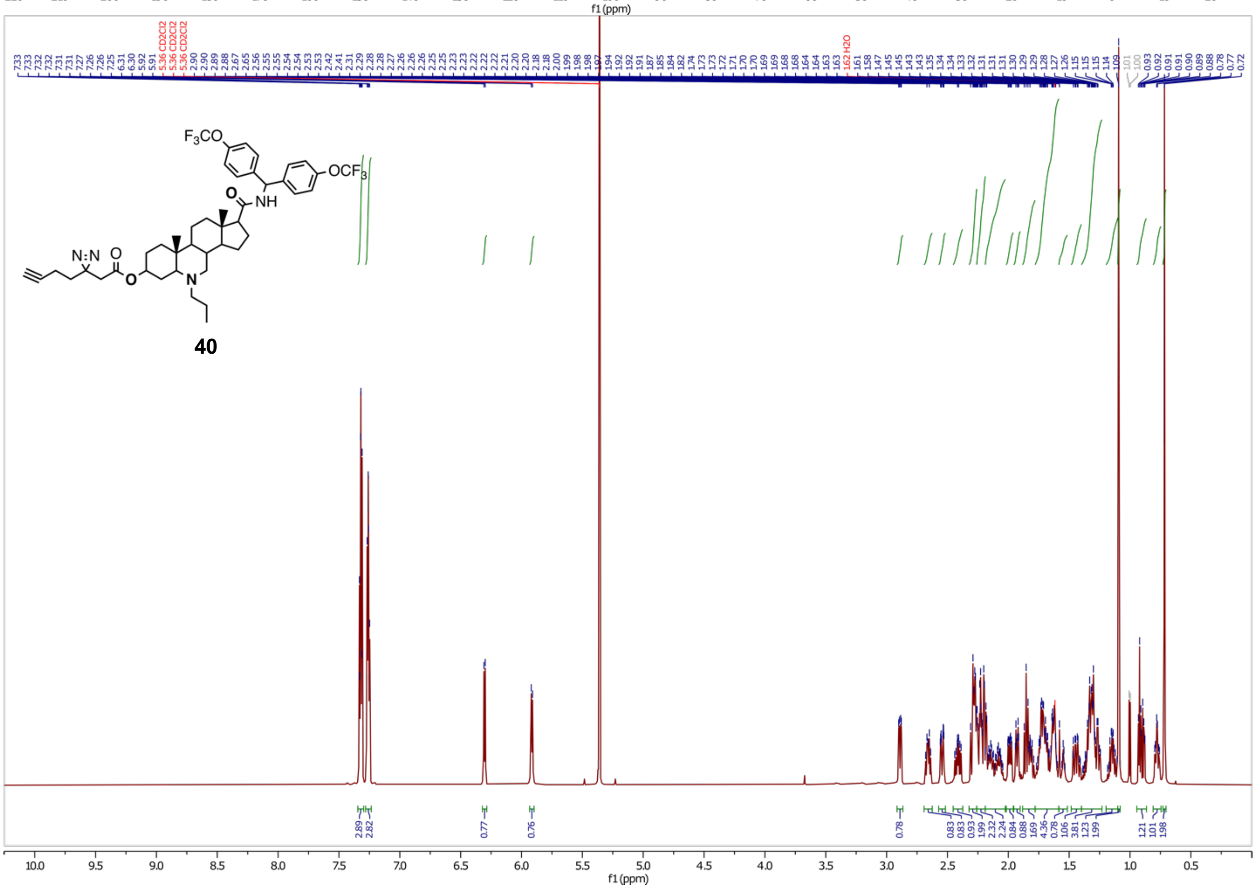
40
f1 (ppm)

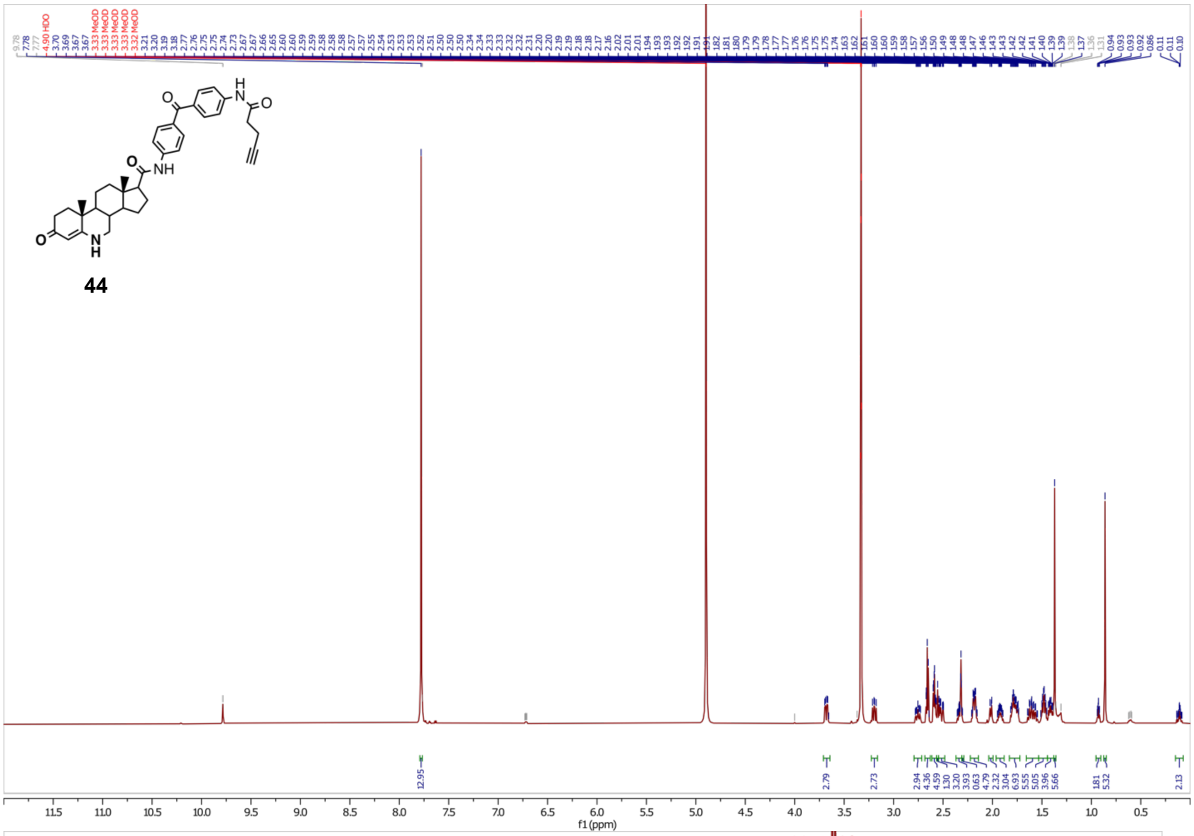

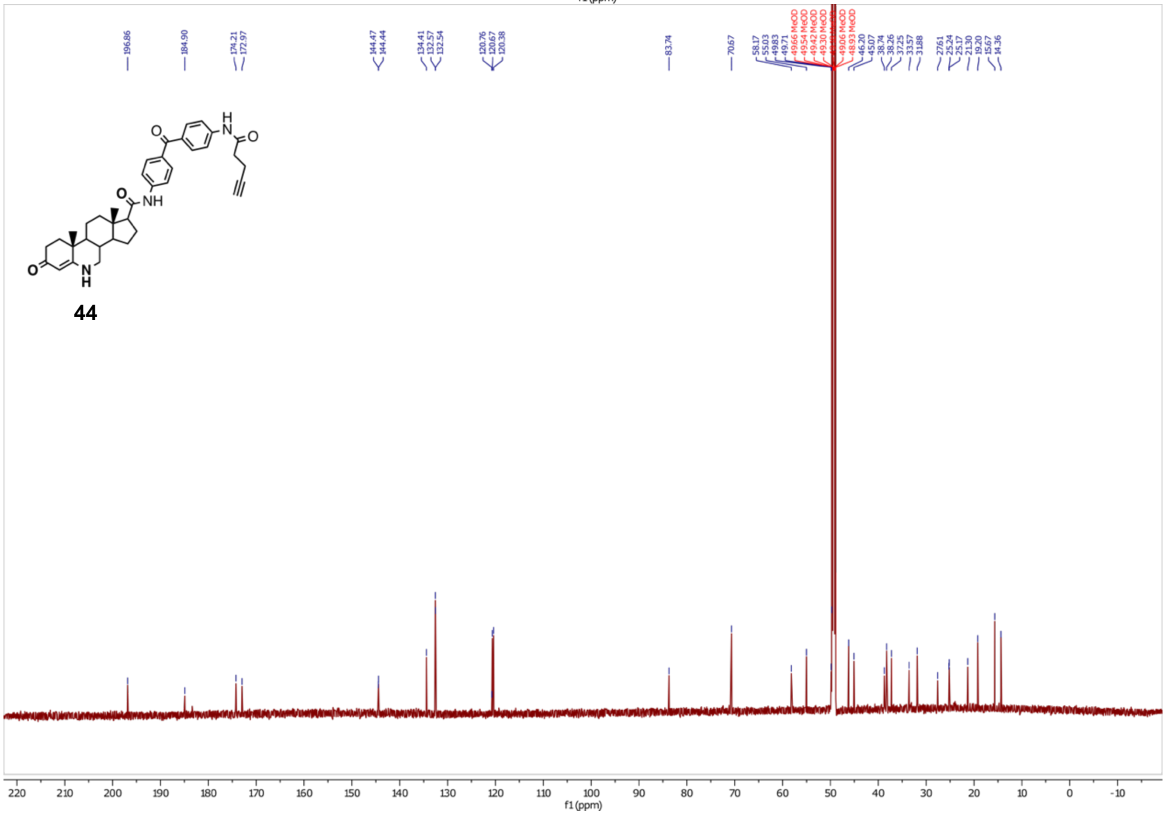

Figure S13. Nuclear magnetic resonance of compounds 40 and 44.

Reference.

(1) Li, S.; Poulton, N. C.; Chang, J. S.; Azadian, Z. A.; DeJesus, M. A.; Ruecker, N.; Zimmerman, M. D.; Eckartt, K. A.; Bosch, B.; Engelhart, C. A.; Sullivan, D. F.; Gengenbacher, M.; Dartois, V. A.; Schnappinger, D.; Rock, J. M. CRISPRi Chemical Genetics and Comparative Genomics Identify Genes Mediating Drug Potency in *Mycobacterium tuberculosis*. *Nat Microbiol* **2022**, *7* (6), 766-779. DOI: 10.1038/s41564-022-01130-y.
